# Hemorrhoidal disease and its genetic association with depression, bipolar disorder, anxiety disorders, and schizophrenia: a bidirectional mendelian randomization study

**DOI:** 10.1186/s40246-024-00588-7

**Published:** 2024-03-21

**Authors:** Zhiguang Huang, Jian Huang, Chun Kai Leung, Casper JP Zhang, Babatunde Akinwunmi, Wai-Kit Ming

**Affiliations:** 1 Department of Infectious Diseases and Public Health, Jockey Club College of Veterinary Medicine and Life Sciences, City University of Hong Kong, Hong Kong SAR, China; 2 Singapore Institute for Clinical Sciences (SICS), Agency for Science, Technology and Research (A*STAR), Singapore, Singapore; 3 Department of Public and International Affairs, City University of Hong Kong, Hong Kong SAR, China; 4 School of Public Health, The University of Hong Kong, Hong Kong SAR, China; 5 Maternal-Fetal Medicine Unit, Brigham and Women’s Hospital, Harvard Medical School, Boston, MA USA

**Keywords:** Hemorrhoidal disease, Mental illness, Mendelian randomization

## Abstract

**Background:**

Hemorrhoids and psychiatric disorders exhibit high prevalence rates and a tendency for relapse in epidemiological studies. Despite this, limited research has explored their correlation, and these studies are often subject to reverse causality and residual confounding. We conducted a Mendelian randomization (MR) analysis to comprehensively investigate the association between several mental illnesses and hemorrhoidal disease.

**Methods:**

Genetic associations for four psychiatric disorders and hemorrhoidal disease were obtained from large consortia, the FinnGen study, and the UK Biobank. Genetic variants associated with depression, bipolar disorder, anxiety disorders, schizophrenia, and hemorrhoidal disease at the genome-wide significance level were selected as instrumental variables. Screening for potential confounders in genetic instrumental variables using PhenoScanner V2. Bidirectional MR estimates were employed to assess the effects of four psychiatric disorders on hemorrhoidal disease.

**Results:**

Our analysis revealed a significant association between genetically predicted depression and the risk of hemorrhoidal disease (IVW, OR=1.20,95% CI=1.09 to 1.33, *P* <0.001). We found no evidence of associations between bipolar disorder, anxiety disorders, schizophrenia, and hemorrhoidal disease. Inverse MR analysis provided evidence for a significant association between genetically predicted hemorrhoidal disease and depression (IVW, OR=1.07,95% CI=1.04 to 1.11, *P* <0.001).

**Conclusions:**

This study offers MR evidence supporting a bidirectional causal relationship between depression and hemorrhoidal disease.

**Supplementary Information:**

The online version contains supplementary material available at 10.1186/s40246-024-00588-7.

## Introduction

Hemorrhoids, characterized by the development of elongated, dilated blood vessels and surrounding supporting tissue within the anal canal, are among the most prevalent anal diseases [1]. They are typically classified as internal or external, depending on their location. Internal hemorrhoids originate above the dentate line and are covered by columnar epithelium, while external hemorrhoids emerge below the dentate line and are covered by squamous epithelium [2–4]. Hemorrhoidal disease, a consequence of anal cushion prolapse, often results in pain and bleeding. With approximately 3.3 million outpatient visits, it ranks as the fourth most common gastrointestinal diagnosis in the United States [5]. Pathogenesis of hemorrhoids involves the weakening of the anal cushion, leading to internal sphincter spasms and hemorrhoid prolapse [6]. Several factors contribute to the development of hemorrhoids, including chronic mental stress, insufficient fiber intake, prolonged bathroom visits, constipation, diarrhea, ascites, and pelvic mass lesions [7]. Although not life-threatening, hemorrhoids can significantly impair patients’ quality of life due to various symptoms such as anal bleeding, pain, and itching.

The relationship between psychological stress and gut health has been extensively studied. For example, some studies have shown that long-term psychological stress may have an impact on intestinal function, such as triggering gastrointestinal symptoms and increasing the risk of irritable bowel syndrome and inflammatory bowel disease [8–10]. However, there is currently insufficient evidence of potential relationships between psychiatric disorders and hemorrhoids. While observational studies have identified a possible link between stress and hemorrhoids [11–13], the causal relationship between psychiatric disorders and hemorrhoidal disease risk remains unclear due to limitations inherent in observational studies, such as residual confounding and reverse causality.

Mendelian randomization (MR) is a method that employs genetic variation as an instrumental variable (IV) to establish the relationship between exposure and outcome [14]. Since genetic variants are randomly assigned at conception and are independent of environmental factors, MR is less susceptible to confounding compared to conventional observational studies [15]. Additionally, as genotypes are not influenced by disease states, MR minimizes the risk of reverse causality. The MR analysis relies on three key assumptions:

(1) The genetic variant, functioning as an IV, demonstrates a strong correlation with the exposure.

(2) The IV is independent of confounders.

(3) The genetic variant affects the target outcome specifically and only in the presence of independent exposure factors, not in the presence of other factors.

This study is designed to explore the potential bidirectional causal relationship between hemorrhoids and various psychiatric disorders. By delving into this potential link, our research aims to enhance the understanding of how psychiatric conditions might be interconnected with hemorrhoids. This insight could lead to innovative approaches in clinical practice, significantly improving the overall treatment outcomes for patients. Additionally, the findings from this study are expected to elevate public awareness about the importance of integrated treatments for both physical and mental health. This could be a catalyst for further medical research and advancements in clinical methodologies, ultimately benefiting patient care on a broader scale.

## Methods

**Fig. 1:**
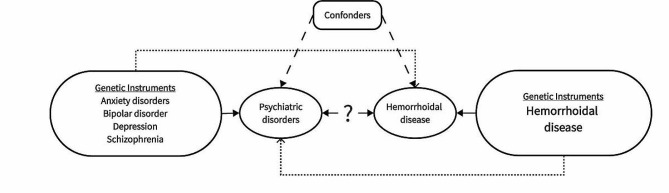
Schematic representation of bidirectional Mendelian randomization study design

Figure 1 illustrates the relationship between four psychiatric disorder phenotypes and hemorrhoidal disease. We conducted a bidirectional MR study to examine the causal relationship between depression, bipolar disorder, anxiety disorders, schizophrenia, and hemorrhoidal disease.

### Data sources for psychiatric disorders

We obtained summary statistics for anxiety disorders from a genome-wide association study (GWAS) conducted by the FinnGen biobank [16] (*n* = 346,542). Summary statistics for bipolar disorder were sourced from a GWAS study conducted by the Psychiatric Genomics Consortium (PGC) Bipolar Disorder Working Group of the Psychiatric Genomics Consortium [17] (*n* = 413,466); the study was a meta-analysis of 57 bipolar disorder cohorts collected in Europe, North America, and Australia, all of which consisted of individuals of European descent. While for depression were obtained from a GWAS meta-analysis study [18] (*n* = 180,866) conducted by the Psychiatric Genomics Consortium [19], which also included new findings from the initial release of UK Biobank (UKB) data [20] and the Resource for Genetic Epidemiology Research on Aging (GERA) cohort (database of Genotypes and Phenotypes (dbGaP), phs000674.v1.p1). The summary statistics for schizophrenia was obtained from the FinnGen biobank [16] (*n* = 405,094). Anxiety disorders, treated as a binary variable, included 301,879 control and 44,663 cases. As defined in the International Classification of Diseases (ICD-10) [21], anxiety disorders encompass various phobic disorders such as agoraphobia with (F40.00) or without (F40.01) panic disorder, social phobia (F40.1), and specific phobias (F40.2), in addition to other types of anxiety disorders. Bipolar disorder was treated as a binary variable, comprising 41,917 control and 371,549 cases. Characterized by recurrent episodes of mania and depression, bipolar disorder (BD) is a severe neuropsychiatric condition that can impact thought processes, perception, emotions, and social behavior. Depression was treated as a binary variable, including 164,574 control and 32,942 cases, with data from this study encompassing varying degrees of depression. Schizophrenia was treated as a binary variable, consisting of 398,386 controls and 6,708 cases. Schizophrenia is a chronic and severe mental disorder with a high heritability rate (64-81%) [22, 23]. In our study, participants in all data sources for psychiatric disorders are European. We selected instrumental variables and obtained summary statistics for the association between genetic variants and psychiatric disorder-related phenotypes using recent GWAS studies as listed in Supplementary Table 1.

### Data sources for hemorrhoidal disease

We acquired summary statistics for hemorrhoidal disease from a meta-analysis GWAS study [24] involving a total of 944,133 participants, utilizing data from five extensive population-based cohorts: 23andMe [25], UK Biobank [26], Estonian Genome Centre at the University of Tartu [27], Michigan Genomics Initiative [28], and Genetic Epidemiology Research on Aging (GERA) [29]. Hemorrhoidal disease was treated as a binary variable, including 725,213 control and 218,920 cases. Consistent with psychiatric disorders, participants in all hemorrhoidal disease data sources were European. We selected instrumental variables and obtained summary data to assess the relationship between genetic variants and hemorrhoidal disease-related phenotypes using several recent GWAS studies listed in Supplementary Table 1.

### Statistical analysis

To investigate the causal relationship between depression, bipolar disorder, anxiety disorders, schizophrenia, and hemorrhoidal disease for each pair of traits, we conducted a bidirectional MR analysis. We selected the genetic instruments for the exposure of interest using a genome-wide significant threshold ($P < 5 \times10^{-8}$). Except for depression and schizophrenia, due to the insufficient number of SNPs obtained, we used a threshold of ${P < 1\times10^{-5}}$. We applied a strong linkage disequilibrium (LD) criterion ($r^{2}=0.001, kb=10,000$) and calculated the F-statistics of the instrumental variables to evaluate weak instrumental variable bias. We harmonized the genetic instrument-exposure data with genetic instrument-outcome data for the same risk-increasing allele and removed all palindromic variants. Furthermore, to satisfy the assumption of independence, we searched SNPs already obtained through PhenoScanner V2 (http://www.phenoscanner.medschl.cam.ac.uk/) [30] to find potential confounders (e.g., high Body Mass Index, pregnancy) [31] may influence the association between psychiatric disorders and hemorrhoids. We noted the possible overlap between samples for hemorrhoidal disease and depression. Burgess et al. [32] suggested that the proportion of sample overlap should be calculated based on the most extensive data set. Therefore, the sample overlap between hemorrhoidal disease and depression did not exceed 17.2% (162,286/944,133). Consequently, we use a web tool (https://sb452.shinyapps.io/overlap/) [32] to evaluate the bias caused by sample overlap. The results show that the bias due to sample overlap is negligible. All calculated deviations of *β* estimates are less than the absolute value of 0.001.

Consistent with previous studies [33], the F-statistic measures instrument strength related to the proportion of variance in the phenotype explained by the formula$F = {{{\beta ^2}_{\exp osure}} \mathord{\left/{\vphantom {{{\beta ^2}_{\exp osure}} {S{E^2}_{\exp osure}}}} \right.\kern-\nulldelimiterspace} {S{E^2}_{\exp osure}}}$. To minimize the potential impact of weak instrument bias when using genotype data, we performed instrument filtering for all exposures with an F-statistic threshold of > 10. We employed the random effect inverse variance weighted (IVW) model as the primary analysis model and the weighted median method [34], the simple mode method [35], the weighted mode method [36], and the MR-Egger method [37] as supplementary methods. The IVW approach provides the most accurate estimates if all SNPs are valid instruments. Additionally, we conducted sensitivity tests using weighted median and MR-Egger regression methods to assess the validity of instruments and investigate influences of potential pleiotropic effects. We conducted Cochran Q test to evaluate heterogeneity and employed the fixed-effects IVW approach as the primary method if the P-values were greater than 0.05 and no evidence of heterogeneity was found. In case of substantial heterogeneity (*P* < 0.05), we utilized the random-effects IVW approach. We utilized the MR-PRESSO [38] approach to identify likely outlier SNPs and excluded them from the sensitivity analysis. Steiger filtering was employed to reduce the possibility that the genetic instruments could influence the outcome independently of the exposure [39]. To investigate the robustness of our model by excluding one IV at a time, we also performed a “leave-one-out” sensitivity test. To address the issue of multiple comparisons across eight association groups, we applied a Bonferroni correction with a significance threshold of *P* < 0.00625 (i.e., 0.05 divided by 8). The “TwoSampleMR” package conducted all statistical analyses in R version 4.2.1.

## Results

**Fig. 2:**
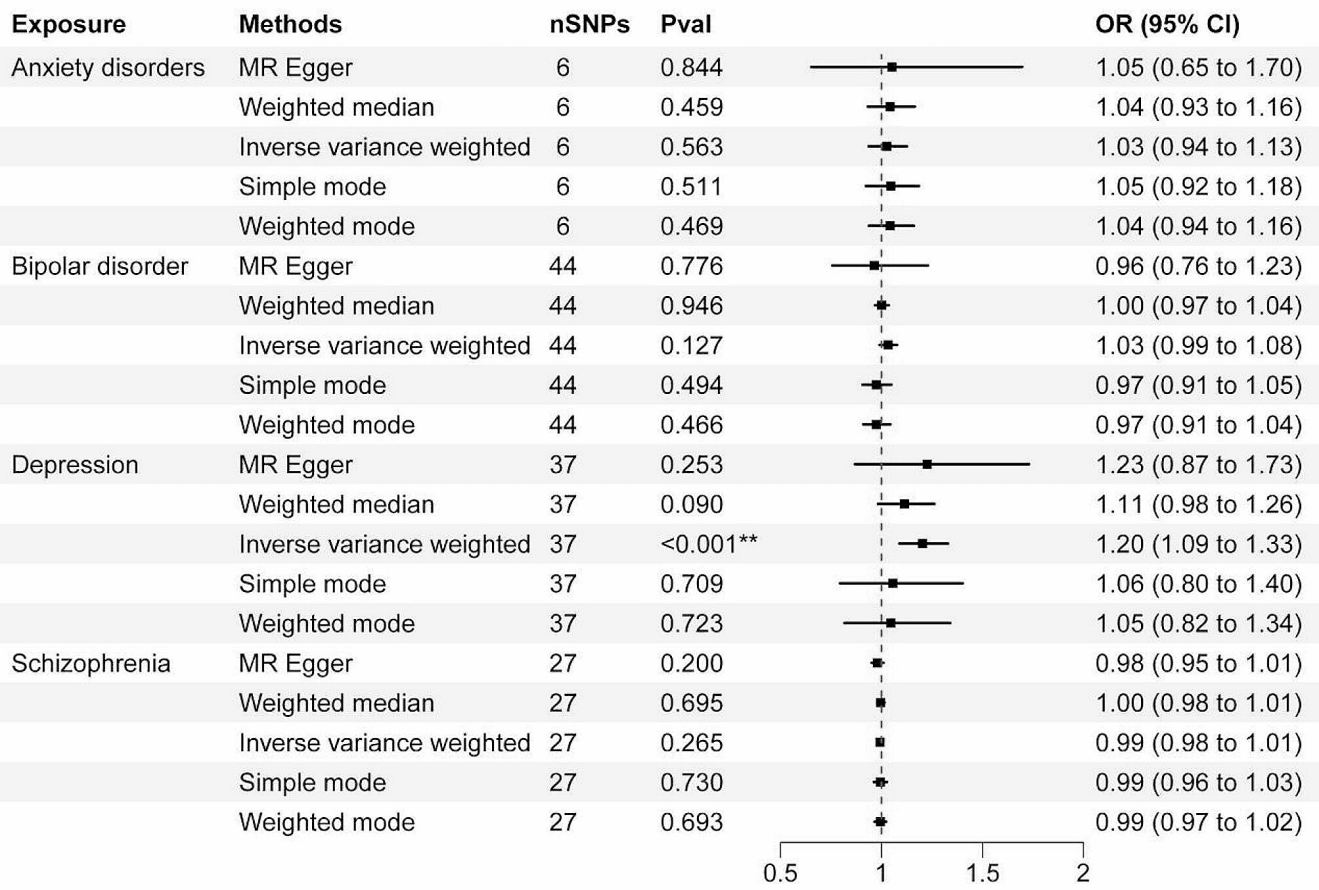
Associations of genetic liability of psychiatric disorders with the hemorrhoidal Disease. *Indicates 0.00625 < *P* < 0.05, **Indicates *P* < 0.00625

**Fig. 3:**
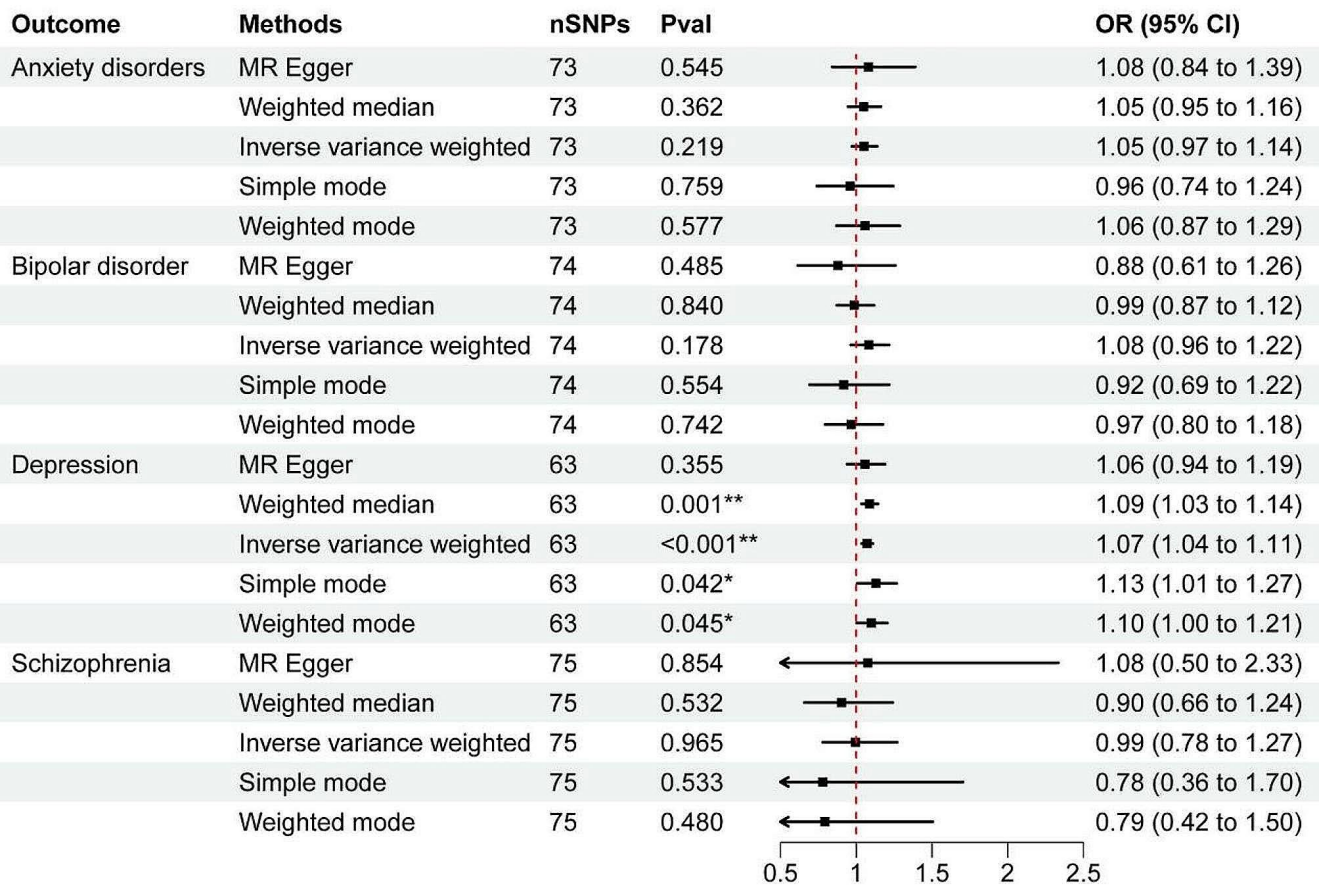
Associations of genetic liability of hemorrhoidal Disease with four psychiatric disorders. *Indicates 0.00625 < *P* < 0.05, **Indicates *P* < 0.00625

In this study, we examined the causal relationships between psychiatric disorders (depression, bipolar disorder, anxiety disorders, schizophrenia) and hemorrhoidal disease by analyzing each pair of traits using bidirectional MR. Detailed information on instrumental variables (i.e., SNPs associated with exposure) varied for depression, bipolar disorder, anxiety disorders, schizophrenia, and hemorrhoidal disease (Table 1; Figs. 2 and 3). Figures S1 and S2 display scatter plots of associations between depression, bipolar disorder, anxiety disorders, schizophrenia, and hemorrhoidal disease. Table 1, S2, and Figures S1 through S18 provide the complete results.

**Table 1:** Two-sample MR result: the causal effect of depression, bipolar disorder, anxiety disorders, schizophrenia, and hemorrhoidal Disease

Exposure	Outcome	Method	N(snp)	β	se	pval	OR (95%CI)	Heterogeneity P value	Intercept p value
Anxiety disorders	Hemorrhoidal Disease	MR Egger	6	0.051	0.243	0.844	1.052(0.653–1.696)	0.006	0.295
Anxiety disorders	Hemorrhoidal Disease	Weighted median	6	0.042	0.057	0.459	1.043(0.933–1.165)	Na	Na
Anxiety disorders	Hemorrhoidal Disease	Inverse variance weighted	6	0.027	0.047	0.563	1.027(0.937–1.126)	0.003	Na
Anxiety disorders	Hemorrhoidal Disease	Simple mode	6	0.045	0.064	0.511	1.046(0.923–1.185)	Na	Na
Anxiety disorders	Hemorrhoidal Disease	Weighted mode	6	0.042	0.054	0.469	1.043(0.938–1.160)	Na	Na
Bipolar disorder	Hemorrhoidal Disease	MR Egger	44	-0.035	0.124	0.776	0.965(0.757–1.230)	< 0.001	0.580
Bipolar disorder	Hemorrhoidal Disease	Weighted median	44	0.001	0.017	0.946	1.001(0.968–1.036)	Na	Na
Bipolar disorder	Hemorrhoidal Disease	Inverse variance weighted	44	0.033	0.021	0.127	1.033(0.991–1.077)	< 0.001	Na
Bipolar disorder	Hemorrhoidal Disease	Simple mode	44	-0.026	0.037	0.494	0.975(0.906–1.049)	Na	Na
Bipolar disorder	Hemorrhoidal Disease	Weighted mode	44	-0.026	0.035	0.466	0.975(0.910–1.044)	Na	Na
Depression	Hemorrhoidal Disease	MR Egger	37	0.204	0.176	0.253	1.226(0.869–1.730)	0.004	0.911
Depression	Hemorrhoidal Disease	Weighted median	37	0.108	0.064	0.090	1.114(0.983–1.262)	Na	Na
Depression	Hemorrhoidal Disease	Inverse variance weighted	37	0.185	0.051	< 0.001	1.203(1.089–1.329)	0.005	Na
Depression	Hemorrhoidal Disease	Simple mode	37	0.054	0.145	0.709	1.056(0.795–1.402)	Na	Na
Depression	Hemorrhoidal Disease	Weighted mode	37	0.045	0.126	0.723	1.046(0.817–1.340)	Na	Na
Schizophrenia	Hemorrhoidal Disease	MR Egger	27	-0.020	0.015	0.200	0.980(0.951–1.010)	0.612	0.363
Schizophrenia	Hemorrhoidal Disease	Weighted median	27	-0.004	0.009	0.695	0.996(0.979–1.015)	Na	Na
Schizophrenia	Hemorrhoidal Disease	Inverse variance weighted	27	-0.007	0.007	0.265	0.993(0.980–1.006)	0.617	Na
Schizophrenia	Hemorrhoidal Disease	Simple mode	27	-0.006	0.017	0.729	0.994(0.962–1.027)	Na	Na
Schizophrenia	Hemorrhoidal Disease	Weighted mode	27	-0.006	0.015	0.693	0.994(0.966–1.023)	Na	Na
Hemorrhoidal Disease	Anxiety disorders	MR Egger	73	0.078	0.128	0.545	1.081(0.841–1.390)	< 0.001	0.869
Hemorrhoidal Disease	Anxiety disorders	Weighted median	73	0.048	0.053	0.362	1.049(0.946–1.163)	Na	Na
Hemorrhoidal Disease	Anxiety disorders	Inverse variance weighted	73	0.050	0.041	0.219	1.052(0.971–1.139)	< 0.001	Na
Hemorrhoidal Disease	Anxiety disorders	Simple mode	73	-0.041	0.132	0.759	0.960(0.741–1.244)	Na	Na
Hemorrhoidal Disease	Anxiety disorders	Weighted mode	73	0.056	0.100	0.577	1.058(0.869–1.288)	Na	Na
Hemorrhoidal Disease	Bipolar disorder	MR Egger	74	-0.129	0.183	0.485	0.879(0.614–1.259)	< 0.001	0.233
Hemorrhoidal Disease	Bipolar disorder	Weighted median	74	-0.013	0.063	0.840	0.987(0.872–1.118)	Na	Na
Hemorrhoidal Disease	Bipolar disorder	Inverse variance weighted	74	0.080	0.059	0.178	1.083(0.964–1.217)	< 0.001	Na
Hemorrhoidal Disease	Bipolar disorder	Simple mode	74	-0.086	0.144	0.554	0.918(0.692–1.218)	Na	Na
Hemorrhoidal Disease	Bipolar disorder	Weighted mode	74	-0.033	0.100	0.742	0.967(0.795–1.177)	Na	Na
Hemorrhoidal Disease	Depression	MR Egger	63	0.056	0.060	0.355	1.058(0.940–1.190)	0.028	0.753
Hemorrhoidal Disease	Depression	Weighted median	63	0.085	0.026	0.001	1.088(1.035–1.144)	Na	Na
Hemorrhoidal Disease	Depression	Inverse variance weighted	63	0.070	0.018	< 0.001	1.072(1.036–1.110)	0.034	Na
Hemorrhoidal Disease	Depression	Simple mode	63	0.122	0.059	0.042	1.130(1.007–1.267)	Na	Na
Hemorrhoidal Disease	Depression	Weighted mode	63	0.096	0.047	0.045	1.100(1.004–1.206)	Na	Na
Hemorrhoidal Disease	Schizophrenia	MR Egger	75	0.073	0.395	0.854	1.076(0.496–2.334)	< 0.001	0.561
Hemorrhoidal Disease	Schizophrenia	Weighted median	75	-0.101	0.162	0.532	0.904(0.658–1.241)	Na	Na
Hemorrhoidal Disease	Schizophrenia	Inverse variance weighted	75	-0.005	0.125	0.965	0.995(0.779–1.270)	< 0.001	Na
Hemorrhoidal Disease	Schizophrenia	Simple mode	75	-0.250	0.399	0.533	0.779(0.356–1.704)	Na	Na
Hemorrhoidal Disease	Schizophrenia	Weighted mode	75	-0.232	0.327	0.480	0.793(0.418–1.505)	Na	Na

Remark: N (SNPs) represents the number of SNPs in depression, bipolar disorder, anxiety disorders, schizophrenia, and hemorrhoidal Disease. *β*, effect size. OR, odds ratio. CI, confidence intervals

### Sensitivity analysis

We observed substantial heterogeneity (Table 1) in the effect estimates for hemorrhoidal disease and several exposures (depression, bipolar disorder, anxiety disorders). Specific exposure phenotypes confirmed this heterogeneity, with weighted median analyses yielding opposite results compared to the IVW approach. In the subsequent pleiotropic analysis (Table 1), there was no evidence of pleiotropic effects for all exposures and outcomes for our SNPs. We used MR-PRESSO (Table S2) to detect and remove outliers. After removing outliers, our results are more robust and reliable. When performing the leave-one-out analysis (Figures S3 to S10), we found a small number of SNPs whose orientation was inconsistent with the overall orientation. However, these SNPs passed all our other sensitivity tests, proving they were not outliers. Funnel plots (Figures S11 to S18) were used to visualize the MR effects of hemorrhoidal disease and four psychiatric disorders, as determined by IVW and MR-Egger regression analyses. All SNPs (black dots) are symmetrically evenly distributed on both sides of the median axis (estimated by IVW and MR-Egger regression). There are no outliers significantly off the central axis, indicating that our fitting results were robust.

### Mendelian randomization analysis

Figure S1 displays the scatter plot of effect sizes for each single-nucleotide polymorphism (SNP) on anxiety disorders, bipolar disorder, depression, schizophrenia, and hemorrhoidal disease risk. Figure S2 illustrates the scatter plot of effect sizes for each SNP on hemorrhoidal disease and anxiety disorders risk, bipolar disorder risk, depression risk, and schizophrenia risk. No causal relationship was found between anxiety disorders, bipolar disorder, schizophrenia, and hemorrhoidal disease in bidirectional MR approaches. Genetically higher hemorrhoidal disease risk is significantly associated with depression (IVW, $\text{O}\text{R}=1.20, 95\text{\%} \text{C}\text{I}=1.09 \text{t}\text{o} 1.33, P <0.001)$, while hemorrhoidal disease can also increase the risk of depression (IVW,$\text{O}\text{R}=1.07, 95\text{\%} \text{C}\text{I}=1.04 \text{t}\text{o} 1.11, P <0.001)$. MR findings using IVW, weighted median, simple mode, weighted median, and MR-Egger were consistent.

## Discussion

Our bidirectional MR study, utilizing comprehensive data from GWAS, uncovered a causal relationship between depression and hemorrhoidal disease, substantiating a positive association. However, no discernible causal relationship was found between hemorrhoidal disease risk and the other three investigated psychiatric disorders (bipolar disorder, anxiety disorders, schizophrenia).

These findings are consistent with prior observational studies. For example, a Korean National Health and Nutrition Examination Survey reported a significantly increased risk of developing hemorrhoids among individuals self-reporting depression or diagnosed with depression by a physician [11]. The association between depression and hemorrhoids may be explained by an increased risk of eating disorders and reduced physical activity among those with depression [40, 41]. However, due to the cross-sectional design of these studies, the temporal sequence between hemorrhoids and risk factors remains uncertain, leaving the causality of the observed associations unclear. A prospective controlled study examining psychiatric symptoms in benign anorectal disorders (including hemorrhoids) revealed that patients exhibited higher levels of psychiatric symptoms compared to the control group [42]. These studies suggest that emotional state fluctuations may play a significant role in benign anorectal disorder development. Nevertheless, it is important to note that both studies had small sample sizes and non-homogeneous patient and control groups. Overcoming the limitations of previous observational studies, our MR investigation reliably confirms a bidirectional association between depression and hemorrhoidal disease.

While observational studies have identified anxiety disorders, bipolar disorder, and other psychiatric disorders as complications in patients with hemorrhoids [43–45], no studies have reported an independent causal relationship between these conditions. Our MR study found no significant causal relationship between hemorrhoidal disease and anxiety, bipolar disorder, or schizophrenia. Simultaneously, our research highlights a potential bidirectional causal relationship between hemorrhoidal disease and depression, emphasizing the importance of mental health in preventing disease deterioration for patients with hemorrhoidal disease. Additionally, to reduce the incidence of hemorrhoidal disease, increased attention should be given to the personal hygiene and living habits of patients with depression.

Mental health conditions, such as depression and anxiety, can significantly impact digestive health [46]. When the brain receives stress input, multiple pathways of the autonomic nervous system and the hypothalamic-pituitary-adrenal axis (HPA axis) are activated. These pathways come from different sources of stress, which may lead to changes in the brain-gut axis and ultimately lead to a variety of gastrointestinal disease [47]. Animal and clinical studies have also shown that stress can lead to dysbiosis. The microbiota communicates with the brain-gut axis through mucosal cells, immune cells, and nerve terminals [48]. Stress-induced dysbiosis affects the host-microbiota and gastrointestinal health through modulation of the neuro-immune-endocrine system [49–51]. Research has shown that individuals with mental health issues are more likely to experience digestive problems, including constipation, which can lead to hemorrhoids [52–54]. Moreover, those with mental health problems may be more inclined to engage in unhealthy habits, such as smoking or consuming alcohol [55], both of which increase the risk of hemorrhoids [56]. Conversely, the pain and discomfort caused by hemorrhoids can contribute to depressive or anxious thoughts. Furthermore, due to the discomfort or embarrassment associated with their condition, individuals with hemorrhoids may avoid social situations [57], potentially resulting in feelings of loneliness and isolation that can exacerbate preexisting mental health problems. Addressing both conditions’ prevention and treatment is crucial, with a focus on underlying causes, such as medical conditions or lifestyle factors. For example, lifestyle adjustments like increased fiber intake or regular exercise can help alleviate constipation, reducing the likelihood of developing hemorrhoids. Treatment for underlying mental health conditions, such as depression or anxiety, can also alleviate symptoms.

Additionally, treatment methods for patients with hemorrhoidal diseases should be considered. Conservative medical treatment for hemorrhoid disease typically involves lifestyle and dietary modifications [5]. However, hemorrhoids are prone to relapse, and single conservative treatments may not suffice for severe patients. Most patients are also reluctant to undergo hemorrhoid surgery [58], which can lead to disease delay and deterioration, further impacting their mental health. Therefore, it is essential to persuade patients with severe conditions to undergo timely surgical treatment to reduce their risk of depression.

Our study has several strengths, including being the first MR analysis of hemorrhoidal diseases and several psychiatric disorders. We also utilized a GWAS meta-analysis of multiple large population cohorts, which increased our data size and made the results more robust. Furthermore, by using single nucleotide polymorphisms (SNPs) as instrumental variables, MR studies reduce the risk of reverse causality and confounders common in observational studies. The data sources for the included exposures and outcomes were mostly obtained from different GWAS, reducing the possibility of sample overlap.

Our study also has several limitations. First, we used aggregated data from multiple databases. Due to the lack of more specific information, it is impossible to conduct stratified analyses, such as by gender and age. Second, our analysis was limited to European populations, so whether the observed associations can be generalized to other populations remains to be determined. Third, the samples of depression and hemorrhoids partially overlapped, with some individuals included in both the outcome and exposure samples. However, considering the larger sample size for hemorrhoids compared to depression, the overlap ratio is small (≤ 17.20%). Calculations show that the estimation bias resulting from this sample overlap is negligible. Fourth, our study’s selection of genetic variants was based on the statistical significance approach, which may introduce the risk of the “winner’s curse.” This phenomenon entails an overestimation of effect sizes due to random error. We took rigorous measures to address this potential limitation by conducting statistical corrections and implementing various sensitivity analyses. Specifically, we performed extensive sensitivity analyses to select each instrumental variable meticulously. To evaluate the strength of these instrumental variables, we calculated the F value. In our study, the average F value of the selected SNPs was determined to be 45.02, indicating the instrumental variables we selected are relatively solid. A further potential limitation is that our MR research only reveals the possible causal relationship between depression and hemorrhoids from a genetic and statistical perspective, necessitating further clinical research to elucidate the biological pathogenic mechanisms.

Our study provides initial evidence of a possible causal connection between depression and hemorrhoidal disease. However, further research must investigate the relationship and specific mechanisms underlying these conditions. We look forward to future studies that will reveal the causal pathways linking depression and hemorrhoids, thus providing scientific backing for the creation of more successful intervention methods to reduce patient distress and enhance their quality of life.

## Conclusions

In conclusion, this study investigated hemorrhoid-related psychiatric disorders using a series of comprehensive MR analyses. We initially observed a bidirectional association between genetically predicted risk of depression and hemorrhoidal disease using univariate MR. We did not find evidence for a causal relationship between hemorrhoidal disease and anxiety, bipolar disorder, or schizophrenia. This study highlights the importance of considering both mental and physical health factors in the prevention and treatment of hemorrhoidal disease and depression, emphasizing the need for a comprehensive approach to patient care.

### Electronic supplementary material

Below is the link to the electronic supplementary material.

Supplementary Material 1

## Data Availability

The summary data for Hemorrhoidal Disease are available at https://gwas.mrcieu.ac.uk/datasets/ebi-a-GCST90014033/ The summary data for Depression are available at https://gwas.mrcieu.ac.uk/datasets/ebi-a-GCST003769/ The summary data for Bipolar Disorder are available at https://gwas.mrcieu.ac.uk/datasets/ieu-b-5110/ The summary data for Anxiety Disorders are available at https://r10.finngen.fi/ The summary data for Schizophrenia are available at https://r10.finngen.fi/
