# Supplementary material for: Hemorrhoidal disease and its genetic association with depression, bipolar disorder, anxiety disorders, and schizophrenia: a bidirectional mendelian randomization study
Source: Hum Genomics. 2024 Mar 21;18:27. doi: 10.1186/s40246-024-00588-7 (PMC10956248; doi:10.1186/s40246-024-00588-7)
Supplement: Supplementary file 1 — Supplementary Material 1 [file 40246_2024_588_MOESM1_ESM.docx]

**Supplementary Tables**

**Supplementary** **Table** **1**: IVs related to exposures by screening LD

| Exposure | Outcome | SNP | effect allele | other allele | β | P | se | F |
| --- | --- | --- | --- | --- | --- | --- | --- | --- |
| Anxiety disorders | Hemorrhoidal Disease | rs10092618 | A | G | 0.058 | 8.07E-12 | 0.008 | 46.75 |
| Anxiety disorders | Hemorrhoidal Disease | rs145274568 | A | G | 0.071 | 2.68E-08 | 0.013 | 30.92 |
| Anxiety disorders | Hemorrhoidal Disease | rs145281382 | T | C | 0.106 | 3.50E-10 | 0.017 | 39.37 |
| Anxiety disorders | Hemorrhoidal Disease | rs1480567 | C | T | 0.048 | 2.46E-08 | 0.009 | 31.09 |
| Anxiety disorders | Hemorrhoidal Disease | rs62099231 | A | G | 0.043 | 1.56E-08 | 0.008 | 31.97 |
| Anxiety disorders | Hemorrhoidal Disease | rs77683334 | T | C | -0.121 | 5.06E-09 | 0.021 | 34.17 |
| Bipolar disorder | Hemorrhoidal Disease | rs10043984 | T | C | 0.059 | 3.71E-08 | 0.011 | 30.15 |
| Bipolar disorder | Hemorrhoidal Disease | rs10255167 | A | G | 0.066 | 1.60E-08 | 0.012 | 31.66 |
| Bipolar disorder | Hemorrhoidal Disease | rs10737496 | T | C | -0.054 | 7.17E-09 | 0.009 | 33.25 |
| Bipolar disorder | Hemorrhoidal Disease | rs10866641 | C | T | -0.063 | 2.79E-11 | 0.009 | 44.35 |
| Bipolar disorder | Hemorrhoidal Disease | rs10994415 | C | T | 0.118 | 1.14E-11 | 0.017 | 46.07 |
| Bipolar disorder | Hemorrhoidal Disease | rs112481526 | G | A | 0.063 | 1.86E-09 | 0.011 | 36.11 |
| Bipolar disorder | Hemorrhoidal Disease | rs113779084 | A | G | 0.075 | 1.42E-13 | 0.010 | 54.79 |
| Bipolar disorder | Hemorrhoidal Disease | rs11764361 | G | A | -0.061 | 3.47E-09 | 0.010 | 34.97 |
| Bipolar disorder | Hemorrhoidal Disease | rs12575685 | A | G | 0.065 | 1.24E-10 | 0.010 | 41.67 |
| Bipolar disorder | Hemorrhoidal Disease | rs12668848 | A | G | -0.057 | 1.90E-09 | 0.010 | 36.01 |
| Bipolar disorder | Hemorrhoidal Disease | rs13044225 | G | A | 0.055 | 8.50E-09 | 0.010 | 33.15 |
| Bipolar disorder | Hemorrhoidal Disease | rs1487445 | T | C | 0.074 | 1.48E-15 | 0.009 | 63.65 |
| Bipolar disorder | Hemorrhoidal Disease | rs17183814 | A | G | -0.103 | 2.68E-08 | 0.019 | 30.94 |
| Bipolar disorder | Hemorrhoidal Disease | rs174592 | G | A | 0.072 | 9.92E-14 | 0.010 | 55.10 |
| Bipolar disorder | Hemorrhoidal Disease | rs2126180 | A | G | 0.057 | 1.62E-09 | 0.009 | 36.26 |
| Bipolar disorder | Hemorrhoidal Disease | rs2273738 | T | C | 0.092 | 1.63E-11 | 0.014 | 45.46 |
| Bipolar disorder | Hemorrhoidal Disease | rs228768 | T | G | -0.064 | 2.83E-10 | 0.010 | 39.86 |
| Bipolar disorder | Hemorrhoidal Disease | rs2336147 | C | T | -0.068 | 3.61E-13 | 0.009 | 52.99 |
| Bipolar disorder | Hemorrhoidal Disease | rs237460 | T | C | 0.055 | 4.25E-09 | 0.009 | 34.61 |
| Bipolar disorder | Hemorrhoidal Disease | rs2693698 | G | A | 0.053 | 1.96E-08 | 0.009 | 31.79 |
| Bipolar disorder | Hemorrhoidal Disease | rs28455634 | A | G | -0.063 | 2.63E-10 | 0.010 | 40.23 |
| Bipolar disorder | Hemorrhoidal Disease | rs28565152 | A | G | 0.067 | 1.96E-09 | 0.011 | 35.89 |
| Bipolar disorder | Hemorrhoidal Disease | rs2953928 | A | G | 0.116 | 6.25E-09 | 0.020 | 33.70 |
| Bipolar disorder | Hemorrhoidal Disease | rs35306827 | A | G | -0.066 | 3.56E-09 | 0.011 | 34.62 |
| Bipolar disorder | Hemorrhoidal Disease | rs35958438 | A | G | -0.064 | 3.83E-08 | 0.012 | 30.11 |
| Bipolar disorder | Hemorrhoidal Disease | rs41315395 | A | C | 0.072 | 1.48E-08 | 0.013 | 32.06 |
| Bipolar disorder | Hemorrhoidal Disease | rs4447398 | C | A | -0.082 | 2.61E-09 | 0.014 | 35.48 |
| Bipolar disorder | Hemorrhoidal Disease | rs4619651 | A | G | -0.066 | 4.78E-11 | 0.010 | 42.83 |
| Bipolar disorder | Hemorrhoidal Disease | rs4790841 | T | C | 0.073 | 3.14E-08 | 0.013 | 30.50 |
| Bipolar disorder | Hemorrhoidal Disease | rs5758064 | C | T | -0.052 | 2.01E-08 | 0.009 | 31.75 |
| Bipolar disorder | Hemorrhoidal Disease | rs6104027 | G | A | -0.060 | 1.93E-10 | 0.010 | 40.30 |
| Bipolar disorder | Hemorrhoidal Disease | rs61554907 | T | G | 0.087 | 1.64E-08 | 0.015 | 31.77 |
| Bipolar disorder | Hemorrhoidal Disease | rs62581014 | T | C | 0.065 | 2.77E-08 | 0.012 | 31.05 |
| Bipolar disorder | Hemorrhoidal Disease | rs6806239 | G | T | -0.066 | 2.64E-08 | 0.012 | 30.85 |
| Bipolar disorder | Hemorrhoidal Disease | rs6887473 | A | G | -0.060 | 8.81E-09 | 0.011 | 32.99 |
| Bipolar disorder | Hemorrhoidal Disease | rs6946056 | C | A | 0.053 | 3.66E-08 | 0.010 | 30.08 |
| Bipolar disorder | Hemorrhoidal Disease | rs6954854 | A | G | -0.058 | 5.94E-10 | 0.009 | 38.46 |
| Bipolar disorder | Hemorrhoidal Disease | rs696366 | A | C | -0.052 | 4.46E-08 | 0.009 | 30.13 |
| Bipolar disorder | Hemorrhoidal Disease | rs6992333 | G | A | 0.060 | 1.62E-09 | 0.010 | 36.24 |
| Bipolar disorder | Hemorrhoidal Disease | rs7108878 | G | T | 0.081 | 3.61E-08 | 0.015 | 30.37 |
| Bipolar disorder | Hemorrhoidal Disease | rs7201930 | C | T | 0.059 | 1.89E-08 | 0.010 | 31.75 |
| Bipolar disorder | Hemorrhoidal Disease | rs748455 | C | T | -0.067 | 5.01E-11 | 0.010 | 42.95 |
| Bipolar disorder | Hemorrhoidal Disease | rs7707252 | G | A | 0.057 | 3.64E-08 | 0.010 | 30.24 |
| Bipolar disorder | Hemorrhoidal Disease | rs9834970 | C | T | 0.083 | 6.63E-19 | 0.009 | 79.65 |
| Depression | Hemorrhoidal Disease | rs10011311 | T | C | 0.027 | 8.14E-06 | 0.006 | 20.25 |
| Depression | Hemorrhoidal Disease | rs10106565 | T | C | 0.020 | 5.44E-06 | 0.004 | 25.00 |
| Depression | Hemorrhoidal Disease | rs10233018 | G | A | 0.018 | 1.65E-07 | 0.004 | 20.25 |
| Depression | Hemorrhoidal Disease | rs10471073 | T | C | 0.041 | 7.47E-06 | 0.009 | 20.75 |
| Depression | Hemorrhoidal Disease | rs10809520 | T | C | -0.018 | 6.98E-06 | 0.004 | 20.25 |
| Depression | Hemorrhoidal Disease | rs11587416 | T | C | -0.026 | 3.46E-06 | 0.006 | 18.78 |
| Depression | Hemorrhoidal Disease | rs11636582 | T | G | -0.042 | 6.98E-07 | 0.009 | 21.78 |
| Depression | Hemorrhoidal Disease | rs12515869 | G | T | 0.041 | 5.75E-06 | 0.009 | 20.75 |
| Depression | Hemorrhoidal Disease | rs139560451 | T | C | -0.059 | 2.88E-06 | 0.013 | 20.60 |
| Depression | Hemorrhoidal Disease | rs1431071 | T | G | -0.016 | 9.33E-06 | 0.004 | 16.00 |
| Depression | Hemorrhoidal Disease | rs148701159 | G | A | -0.035 | 3.74E-06 | 0.008 | 19.14 |
| Depression | Hemorrhoidal Disease | rs1690818 | T | C | -0.019 | 4.05E-07 | 0.004 | 22.56 |
| Depression | Hemorrhoidal Disease | rs1877075 | A | G | -0.022 | 2.00E-06 | 0.005 | 19.36 |
| Depression | Hemorrhoidal Disease | rs1961982 | A | G | -0.022 | 2.25E-06 | 0.005 | 19.36 |
| Depression | Hemorrhoidal Disease | rs2017122 | T | C | 0.033 | 2.32E-06 | 0.007 | 22.22 |
| Depression | Hemorrhoidal Disease | rs2327613 | C | T | 0.016 | 7.21E-06 | 0.004 | 16.00 |
| Depression | Hemorrhoidal Disease | rs2721938 | T | C | 0.015 | 7.68E-06 | 0.003 | 25.00 |
| Depression | Hemorrhoidal Disease | rs2927712 | C | A | 0.027 | 7.06E-06 | 0.006 | 20.25 |
| Depression | Hemorrhoidal Disease | rs3891649 | T | C | -0.015 | 6.55E-06 | 0.003 | 25.00 |
| Depression | Hemorrhoidal Disease | rs4292572 | G | T | 0.024 | 7.11E-06 | 0.005 | 23.04 |
| Depression | Hemorrhoidal Disease | rs4810896 | C | A | 0.017 | 9.00E-07 | 0.003 | 32.11 |
| Depression | Hemorrhoidal Disease | rs4878386 | T | C | -0.016 | 7.69E-06 | 0.004 | 16.00 |
| Depression | Hemorrhoidal Disease | rs4942916 | T | C | -0.022 | 9.97E-07 | 0.005 | 19.36 |
| Depression | Hemorrhoidal Disease | rs59659806 | C | T | 0.022 | 6.01E-07 | 0.004 | 30.25 |
| Depression | Hemorrhoidal Disease | rs61731746 | G | T | -0.049 | 5.17E-06 | 0.011 | 19.84 |
| Depression | Hemorrhoidal Disease | rs6458013 | T | C | 0.023 | 1.02E-06 | 0.005 | 21.16 |
| Depression | Hemorrhoidal Disease | rs6828666 | A | G | -0.019 | 6.91E-06 | 0.004 | 22.56 |
| Depression | Hemorrhoidal Disease | rs6992714 | C | T | -0.020 | 9.32E-08 | 0.004 | 25.00 |
| Depression | Hemorrhoidal Disease | rs703632 | A | G | -0.016 | 9.43E-06 | 0.004 | 16.00 |
| Depression | Hemorrhoidal Disease | rs7074335 | C | T | -0.035 | 4.57E-07 | 0.007 | 25.00 |
| Depression | Hemorrhoidal Disease | rs72939513 | A | G | -0.044 | 3.14E-06 | 0.009 | 23.90 |
| Depression | Hemorrhoidal Disease | rs77508728 | T | C | 0.055 | 4.91E-06 | 0.012 | 21.01 |
| Depression | Hemorrhoidal Disease | rs782212 | T | C | -0.018 | 1.23E-06 | 0.004 | 20.25 |
| Depression | Hemorrhoidal Disease | rs7973260 | G | A | -0.031 | 1.78E-09 | 0.005 | 38.44 |
| Depression | Hemorrhoidal Disease | rs79740545 | T | G | 0.024 | 9.89E-06 | 0.005 | 23.04 |
| Depression | Hemorrhoidal Disease | rs889289 | T | C | -0.018 | 7.17E-06 | 0.004 | 20.25 |
| Depression | Hemorrhoidal Disease | rs9427622 | C | T | 0.021 | 4.75E-07 | 0.004 | 27.56 |
| Schizophrenia | Hemorrhoidal Disease | rs113220789 | A | G | 0.396 | 6.28E-06 | 0.088 | 20.40 |
| Schizophrenia | Hemorrhoidal Disease | rs114666746 | T | C | -0.422 | 4.73E-06 | 0.092 | 20.94 |
| Schizophrenia | Hemorrhoidal Disease | rs117755414 | A | G | 0.282 | 8.56E-06 | 0.063 | 19.81 |
| Schizophrenia | Hemorrhoidal Disease | rs12652777 | C | T | -0.120 | 3.32E-06 | 0.026 | 21.62 |
| Schizophrenia | Hemorrhoidal Disease | rs1345660 | T | G | -0.126 | 2.22E-06 | 0.027 | 22.39 |
| Schizophrenia | Hemorrhoidal Disease | rs145866533 | A | G | -0.504 | 1.90E-06 | 0.106 | 22.69 |
| Schizophrenia | Hemorrhoidal Disease | rs17187183 | G | A | 0.124 | 1.62E-06 | 0.026 | 22.99 |
| Schizophrenia | Hemorrhoidal Disease | rs1899272 | C | T | 0.133 | 6.09E-06 | 0.029 | 20.46 |
| Schizophrenia | Hemorrhoidal Disease | rs2280692 | C | T | -0.151 | 2.99E-06 | 0.032 | 21.83 |
| Schizophrenia | Hemorrhoidal Disease | rs2588988 | C | T | 0.173 | 2.70E-06 | 0.037 | 22.02 |
| Schizophrenia | Hemorrhoidal Disease | rs3128405 | A | G | -0.169 | 3.54E-06 | 0.036 | 21.50 |
| Schizophrenia | Hemorrhoidal Disease | rs59665366 | A | C | 0.155 | 3.86E-06 | 0.034 | 21.33 |
| Schizophrenia | Hemorrhoidal Disease | rs6461352 | A | G | 0.126 | 3.28E-06 | 0.027 | 21.65 |
| Schizophrenia | Hemorrhoidal Disease | rs6966281 | T | G | 0.125 | 3.38E-06 | 0.027 | 21.59 |
| Schizophrenia | Hemorrhoidal Disease | rs72827147 | G | T | -0.284 | 1.39E-06 | 0.059 | 23.30 |
| Schizophrenia | Hemorrhoidal Disease | rs73150893 | A | G | 0.195 | 1.56E-06 | 0.041 | 23.07 |
| Schizophrenia | Hemorrhoidal Disease | rs75289223 | T | G | -0.277 | 1.68E-06 | 0.058 | 22.93 |
| Schizophrenia | Hemorrhoidal Disease | rs76019702 | A | G | -2.018 | 8.50E-07 | 0.410 | 24.24 |
| Schizophrenia | Hemorrhoidal Disease | rs76976716 | C | T | 0.188 | 4.13E-06 | 0.041 | 21.21 |
| Schizophrenia | Hemorrhoidal Disease | rs77012313 | C | A | -0.438 | 7.73E-06 | 0.098 | 20.00 |
| Schizophrenia | Hemorrhoidal Disease | rs77192564 | T | C | 0.145 | 6.15E-06 | 0.032 | 20.44 |
| Schizophrenia | Hemorrhoidal Disease | rs7755858 | C | A | -0.128 | 2.54E-06 | 0.027 | 22.14 |
| Schizophrenia | Hemorrhoidal Disease | rs8000488 | T | C | -0.117 | 7.33E-06 | 0.026 | 20.10 |
| Schizophrenia | Hemorrhoidal Disease | rs9538030 | G | T | -0.342 | 9.81E-06 | 0.077 | 19.55 |
| Hemorrhoidal Disease | Anxiety disorders | rs10153716 | G | A | 0.024 | 9.10E-09 | 0.004 | 32.65 |
| Hemorrhoidal Disease | Anxiety disorders | rs10807610 | C | A | 0.037 | 2.30E-12 | 0.005 | 49.26 |
| Hemorrhoidal Disease | Anxiety disorders | rs10956488 | G | A | 0.033 | 1.66E-09 | 0.006 | 36.22 |
| Hemorrhoidal Disease | Anxiety disorders | rs11045079 | G | A | -0.035 | 1.79E-09 | 0.006 | 36.83 |
| Hemorrhoidal Disease | Anxiety disorders | rs11176001 | A | C | -0.084 | 2.48E-46 | 0.006 | 203.18 |
| Hemorrhoidal Disease | Anxiety disorders | rs1156533 | G | A | -0.024 | 1.76E-08 | 0.004 | 31.94 |
| Hemorrhoidal Disease | Anxiety disorders | rs11578225 | A | G | -0.034 | 6.36E-13 | 0.005 | 51.36 |
| Hemorrhoidal Disease | Anxiety disorders | rs11635984 | C | T | -0.029 | 4.14E-13 | 0.004 | 53.66 |
| Hemorrhoidal Disease | Anxiety disorders | rs11770437 | T | C | -0.038 | 3.18E-18 | 0.004 | 74.19 |
| Hemorrhoidal Disease | Anxiety disorders | rs11942410 | T | C | -0.027 | 1.14E-08 | 0.005 | 32.27 |
| Hemorrhoidal Disease | Anxiety disorders | rs12153515 | T | C | 0.037 | 7.33E-10 | 0.006 | 38.48 |
| Hemorrhoidal Disease | Anxiety disorders | rs12654883 | T | C | 0.025 | 1.19E-08 | 0.004 | 32.03 |
| Hemorrhoidal Disease | Anxiety disorders | rs145163454 | C | T | -0.143 | 5.88E-28 | 0.013 | 120.66 |
| Hemorrhoidal Disease | Anxiety disorders | rs1542726 | C | A | -0.028 | 2.53E-12 | 0.004 | 49.70 |
| Hemorrhoidal Disease | Anxiety disorders | rs16831319 | C | T | 0.048 | 9.95E-09 | 0.008 | 32.52 |
| Hemorrhoidal Disease | Anxiety disorders | rs1689549 | C | T | -0.038 | 4.43E-09 | 0.006 | 34.33 |
| Hemorrhoidal Disease | Anxiety disorders | rs17105613 | C | T | -0.025 | 2.67E-09 | 0.004 | 35.43 |
| Hemorrhoidal Disease | Anxiety disorders | rs17293632 | T | C | 0.054 | 6.88E-32 | 0.005 | 139.34 |
| Hemorrhoidal Disease | Anxiety disorders | rs174767 | G | A | 0.023 | 1.45E-08 | 0.004 | 31.47 |
| Hemorrhoidal Disease | Anxiety disorders | rs17824374 | C | T | -0.033 | 3.35E-11 | 0.005 | 44.54 |
| Hemorrhoidal Disease | Anxiety disorders | rs1838392 | G | T | -0.053 | 4.60E-39 | 0.004 | 172.27 |
| Hemorrhoidal Disease | Anxiety disorders | rs1858015 | T | C | 0.028 | 6.09E-11 | 0.004 | 43.18 |
| Hemorrhoidal Disease | Anxiety disorders | rs2060285 | A | C | 0.025 | 4.19E-09 | 0.004 | 34.03 |
| Hemorrhoidal Disease | Anxiety disorders | rs2186797 | C | T | -0.044 | 4.46E-08 | 0.008 | 29.64 |
| Hemorrhoidal Disease | Anxiety disorders | rs2212450 | C | T | 0.027 | 5.43E-11 | 0.004 | 43.37 |
| Hemorrhoidal Disease | Anxiety disorders | rs2327426 | C | T | -0.026 | 2.68E-09 | 0.004 | 35.72 |
| Hemorrhoidal Disease | Anxiety disorders | rs2421206 | G | T | 0.033 | 2.29E-14 | 0.004 | 58.90 |
| Hemorrhoidal Disease | Anxiety disorders | rs2525570 | G | A | 0.025 | 6.79E-10 | 0.004 | 39.06 |
| Hemorrhoidal Disease | Anxiety disorders | rs2555004 | A | G | 0.025 | 5.36E-10 | 0.004 | 39.46 |
| Hemorrhoidal Disease | Anxiety disorders | rs2605097 | A | C | -0.025 | 4.27E-09 | 0.004 | 34.07 |
| Hemorrhoidal Disease | Anxiety disorders | rs2687965 | A | G | -0.023 | 5.91E-09 | 0.004 | 33.64 |
| Hemorrhoidal Disease | Anxiety disorders | rs2832279 | A | C | 0.027 | 4.46E-11 | 0.004 | 44.01 |
| Hemorrhoidal Disease | Anxiety disorders | rs28663472 | T | C | -0.025 | 4.69E-10 | 0.004 | 39.46 |
| Hemorrhoidal Disease | Anxiety disorders | rs3012065 | C | T | 0.024 | 4.13E-08 | 0.004 | 30.38 |
| Hemorrhoidal Disease | Anxiety disorders | rs3253 | T | C | 0.029 | 4.96E-12 | 0.004 | 48.01 |
| Hemorrhoidal Disease | Anxiety disorders | rs34161672 | A | G | -0.030 | 4.18E-12 | 0.004 | 47.42 |
| Hemorrhoidal Disease | Anxiety disorders | rs3757582 | C | T | 0.056 | 1.69E-10 | 0.009 | 41.14 |
| Hemorrhoidal Disease | Anxiety disorders | rs4233681 | C | T | -0.026 | 2.04E-11 | 0.004 | 45.82 |
| Hemorrhoidal Disease | Anxiety disorders | rs4345978 | T | G | -0.034 | 1.20E-13 | 0.005 | 54.95 |
| Hemorrhoidal Disease | Anxiety disorders | rs4423457 | A | G | -0.022 | 3.36E-08 | 0.004 | 30.53 |
| Hemorrhoidal Disease | Anxiety disorders | rs4556017 | T | C | -0.057 | 1.30E-22 | 0.006 | 96.24 |
| Hemorrhoidal Disease | Anxiety disorders | rs4579999 | C | T | -0.029 | 3.32E-13 | 0.004 | 52.20 |
| Hemorrhoidal Disease | Anxiety disorders | rs4679900 | T | C | 0.023 | 9.52E-09 | 0.004 | 33.06 |
| Hemorrhoidal Disease | Anxiety disorders | rs4843407 | A | G | -0.031 | 4.03E-12 | 0.005 | 47.15 |
| Hemorrhoidal Disease | Anxiety disorders | rs4951080 | G | A | 0.026 | 2.81E-09 | 0.004 | 35.17 |
| Hemorrhoidal Disease | Anxiety disorders | rs56269620 | G | A | 0.034 | 2.31E-16 | 0.004 | 66.76 |
| Hemorrhoidal Disease | Anxiety disorders | rs57116599 | A | G | 0.032 | 1.48E-11 | 0.005 | 46.07 |
| Hemorrhoidal Disease | Anxiety disorders | rs58579887 | C | T | 0.028 | 1.67E-11 | 0.004 | 44.76 |
| Hemorrhoidal Disease | Anxiety disorders | rs61026653 | G | A | -0.033 | 7.62E-09 | 0.006 | 32.91 |
| Hemorrhoidal Disease | Anxiety disorders | rs62061554 | A | G | 0.039 | 2.64E-10 | 0.006 | 39.98 |
| Hemorrhoidal Disease | Anxiety disorders | rs62368263 | C | T | -0.044 | 5.91E-15 | 0.006 | 62.02 |
| Hemorrhoidal Disease | Anxiety disorders | rs6462976 | C | T | 0.028 | 1.23E-12 | 0.004 | 51.55 |
| Hemorrhoidal Disease | Anxiety disorders | rs6482359 | G | A | -0.027 | 3.14E-11 | 0.004 | 43.37 |
| Hemorrhoidal Disease | Anxiety disorders | rs6498573 | T | C | 0.042 | 5.20E-12 | 0.006 | 48.30 |
| Hemorrhoidal Disease | Anxiety disorders | rs6723226 | G | A | 0.024 | 1.99E-08 | 0.004 | 31.57 |
| Hemorrhoidal Disease | Anxiety disorders | rs6792493 | G | A | 0.031 | 9.52E-15 | 0.004 | 59.68 |
| Hemorrhoidal Disease | Anxiety disorders | rs6839705 | C | A | -0.028 | 5.05E-12 | 0.004 | 46.97 |
| Hemorrhoidal Disease | Anxiety disorders | rs6867042 | T | C | -0.035 | 6.02E-09 | 0.006 | 33.83 |
| Hemorrhoidal Disease | Anxiety disorders | rs7183672 | G | A | 0.031 | 1.20E-13 | 0.004 | 55.54 |
| Hemorrhoidal Disease | Anxiety disorders | rs72707023 | A | G | -0.039 | 2.37E-13 | 0.005 | 53.32 |
| Hemorrhoidal Disease | Anxiety disorders | rs728327 | C | T | 0.026 | 2.98E-10 | 0.004 | 40.52 |
| Hemorrhoidal Disease | Anxiety disorders | rs755209 | A | G | 0.024 | 3.06E-08 | 0.004 | 30.50 |
| Hemorrhoidal Disease | Anxiety disorders | rs7594056 | G | A | 0.035 | 7.22E-15 | 0.004 | 61.48 |
| Hemorrhoidal Disease | Anxiety disorders | rs7749659 | G | A | -0.027 | 2.91E-09 | 0.005 | 34.71 |
| Hemorrhoidal Disease | Anxiety disorders | rs7795564 | A | G | -0.029 | 3.38E-13 | 0.004 | 54.02 |
| Hemorrhoidal Disease | Anxiety disorders | rs78378222 | G | T | 0.112 | 4.66E-10 | 0.018 | 38.79 |
| Hemorrhoidal Disease | Anxiety disorders | rs7994724 | G | A | -0.035 | 1.75E-17 | 0.004 | 71.63 |
| Hemorrhoidal Disease | Anxiety disorders | rs8106090 | G | A | 0.022 | 3.34E-08 | 0.004 | 30.96 |
| Hemorrhoidal Disease | Anxiety disorders | rs900400 | C | T | -0.035 | 5.02E-18 | 0.004 | 74.82 |
| Hemorrhoidal Disease | Anxiety disorders | rs9306894 | G | A | -0.026 | 2.34E-10 | 0.004 | 39.91 |
| Hemorrhoidal Disease | Anxiety disorders | rs9322356 | A | G | 0.040 | 2.01E-08 | 0.007 | 31.74 |
| Hemorrhoidal Disease | Anxiety disorders | rs9847710 | C | T | 0.040 | 1.23E-23 | 0.004 | 99.50 |
| Hemorrhoidal Disease | Anxiety disorders | rs9853475 | A | G | 0.026 | 1.96E-09 | 0.004 | 35.73 |
| Hemorrhoidal Disease | Bipolar disorder | rs10153716 | G | A | 0.024 | 9.10E-09 | 0.004 | 32.65 |
| Hemorrhoidal Disease | Bipolar disorder | rs10807610 | C | A | 0.037 | 2.30E-12 | 0.005 | 49.26 |
| Hemorrhoidal Disease | Bipolar disorder | rs10838738 | G | A | 0.025 | 1.69E-09 | 0.004 | 36.59 |
| Hemorrhoidal Disease | Bipolar disorder | rs10956488 | G | A | 0.033 | 1.66E-09 | 0.006 | 36.22 |
| Hemorrhoidal Disease | Bipolar disorder | rs11045079 | G | A | -0.035 | 1.79E-09 | 0.006 | 36.83 |
| Hemorrhoidal Disease | Bipolar disorder | rs11176001 | A | C | -0.084 | 2.48E-46 | 0.006 | 203.18 |
| Hemorrhoidal Disease | Bipolar disorder | rs1156533 | G | A | -0.024 | 1.76E-08 | 0.004 | 31.94 |
| Hemorrhoidal Disease | Bipolar disorder | rs11578225 | A | G | -0.034 | 6.36E-13 | 0.005 | 51.36 |
| Hemorrhoidal Disease | Bipolar disorder | rs11635984 | C | T | -0.029 | 4.14E-13 | 0.004 | 53.66 |
| Hemorrhoidal Disease | Bipolar disorder | rs11770437 | T | C | -0.038 | 3.18E-18 | 0.004 | 74.19 |
| Hemorrhoidal Disease | Bipolar disorder | rs11942410 | T | C | -0.027 | 1.14E-08 | 0.005 | 32.27 |
| Hemorrhoidal Disease | Bipolar disorder | rs12153515 | T | C | 0.037 | 7.33E-10 | 0.006 | 38.48 |
| Hemorrhoidal Disease | Bipolar disorder | rs12654883 | T | C | 0.025 | 1.19E-08 | 0.004 | 32.03 |
| Hemorrhoidal Disease | Bipolar disorder | rs13632 | G | A | -0.030 | 6.12E-11 | 0.005 | 43.67 |
| Hemorrhoidal Disease | Bipolar disorder | rs145163454 | C | T | -0.143 | 5.88E-28 | 0.013 | 120.66 |
| Hemorrhoidal Disease | Bipolar disorder | rs1542726 | C | A | -0.028 | 2.53E-12 | 0.004 | 49.70 |
| Hemorrhoidal Disease | Bipolar disorder | rs16831319 | C | T | 0.048 | 9.95E-09 | 0.008 | 32.52 |
| Hemorrhoidal Disease | Bipolar disorder | rs1689549 | C | T | -0.038 | 4.43E-09 | 0.006 | 34.33 |
| Hemorrhoidal Disease | Bipolar disorder | rs17105613 | C | T | -0.025 | 2.67E-09 | 0.004 | 35.43 |
| Hemorrhoidal Disease | Bipolar disorder | rs17293632 | T | C | 0.054 | 6.88E-32 | 0.005 | 139.34 |
| Hemorrhoidal Disease | Bipolar disorder | rs174767 | G | A | 0.023 | 1.45E-08 | 0.004 | 31.47 |
| Hemorrhoidal Disease | Bipolar disorder | rs17824374 | C | T | -0.033 | 3.35E-11 | 0.005 | 44.54 |
| Hemorrhoidal Disease | Bipolar disorder | rs1838392 | G | T | -0.053 | 4.60E-39 | 0.004 | 172.27 |
| Hemorrhoidal Disease | Bipolar disorder | rs1858015 | T | C | 0.028 | 6.09E-11 | 0.004 | 43.18 |
| Hemorrhoidal Disease | Bipolar disorder | rs2060285 | A | C | 0.025 | 4.19E-09 | 0.004 | 34.03 |
| Hemorrhoidal Disease | Bipolar disorder | rs2186797 | C | T | -0.044 | 4.46E-08 | 0.008 | 29.64 |
| Hemorrhoidal Disease | Bipolar disorder | rs2212450 | C | T | 0.027 | 5.43E-11 | 0.004 | 43.37 |
| Hemorrhoidal Disease | Bipolar disorder | rs2327426 | C | T | -0.026 | 2.68E-09 | 0.004 | 35.72 |
| Hemorrhoidal Disease | Bipolar disorder | rs2421206 | G | T | 0.033 | 2.29E-14 | 0.004 | 58.90 |
| Hemorrhoidal Disease | Bipolar disorder | rs2525570 | G | A | 0.025 | 6.79E-10 | 0.004 | 39.06 |
| Hemorrhoidal Disease | Bipolar disorder | rs2555004 | A | G | 0.025 | 5.36E-10 | 0.004 | 39.46 |
| Hemorrhoidal Disease | Bipolar disorder | rs2605097 | A | C | -0.025 | 4.27E-09 | 0.004 | 34.07 |
| Hemorrhoidal Disease | Bipolar disorder | rs2687965 | A | G | -0.023 | 5.91E-09 | 0.004 | 33.64 |
| Hemorrhoidal Disease | Bipolar disorder | rs2832279 | A | C | 0.027 | 4.46E-11 | 0.004 | 44.01 |
| Hemorrhoidal Disease | Bipolar disorder | rs2861709 | G | A | 0.051 | 2.38E-09 | 0.009 | 35.30 |
| Hemorrhoidal Disease | Bipolar disorder | rs28663472 | T | C | -0.025 | 4.69E-10 | 0.004 | 39.46 |
| Hemorrhoidal Disease | Bipolar disorder | rs3012065 | C | T | 0.024 | 4.13E-08 | 0.004 | 30.38 |
| Hemorrhoidal Disease | Bipolar disorder | rs3253 | T | C | 0.029 | 4.96E-12 | 0.004 | 48.01 |
| Hemorrhoidal Disease | Bipolar disorder | rs34161672 | A | G | -0.030 | 4.18E-12 | 0.004 | 47.42 |
| Hemorrhoidal Disease | Bipolar disorder | rs3757582 | C | T | 0.056 | 1.69E-10 | 0.009 | 41.14 |
| Hemorrhoidal Disease | Bipolar disorder | rs4233681 | C | T | -0.026 | 2.04E-11 | 0.004 | 45.82 |
| Hemorrhoidal Disease | Bipolar disorder | rs4345978 | T | G | -0.034 | 1.20E-13 | 0.005 | 54.95 |
| Hemorrhoidal Disease | Bipolar disorder | rs4556017 | T | C | -0.057 | 1.30E-22 | 0.006 | 96.24 |
| Hemorrhoidal Disease | Bipolar disorder | rs4579999 | C | T | -0.029 | 3.32E-13 | 0.004 | 52.20 |
| Hemorrhoidal Disease | Bipolar disorder | rs4679900 | T | C | 0.023 | 9.52E-09 | 0.004 | 33.06 |
| Hemorrhoidal Disease | Bipolar disorder | rs4843407 | A | G | -0.031 | 4.03E-12 | 0.005 | 47.15 |
| Hemorrhoidal Disease | Bipolar disorder | rs4951080 | G | A | 0.026 | 2.81E-09 | 0.004 | 35.17 |
| Hemorrhoidal Disease | Bipolar disorder | rs56269620 | G | A | 0.034 | 2.31E-16 | 0.004 | 66.76 |
| Hemorrhoidal Disease | Bipolar disorder | rs58579887 | C | T | 0.028 | 1.67E-11 | 0.004 | 44.76 |
| Hemorrhoidal Disease | Bipolar disorder | rs61026653 | G | A | -0.033 | 7.62E-09 | 0.006 | 32.91 |
| Hemorrhoidal Disease | Bipolar disorder | rs62061554 | A | G | 0.039 | 2.64E-10 | 0.006 | 39.98 |
| Hemorrhoidal Disease | Bipolar disorder | rs62368263 | C | T | -0.044 | 5.91E-15 | 0.006 | 62.02 |
| Hemorrhoidal Disease | Bipolar disorder | rs6462976 | C | T | 0.028 | 1.23E-12 | 0.004 | 51.55 |
| Hemorrhoidal Disease | Bipolar disorder | rs6482359 | G | A | -0.027 | 3.14E-11 | 0.004 | 43.37 |
| Hemorrhoidal Disease | Bipolar disorder | rs6498573 | T | C | 0.042 | 5.20E-12 | 0.006 | 48.30 |
| Hemorrhoidal Disease | Bipolar disorder | rs6723226 | G | A | 0.024 | 1.99E-08 | 0.004 | 31.57 |
| Hemorrhoidal Disease | Bipolar disorder | rs677355 | A | G | -0.057 | 5.63E-43 | 0.004 | 186.78 |
| Hemorrhoidal Disease | Bipolar disorder | rs6792493 | G | A | 0.031 | 9.52E-15 | 0.004 | 59.68 |
| Hemorrhoidal Disease | Bipolar disorder | rs6839705 | C | A | -0.028 | 5.05E-12 | 0.004 | 46.97 |
| Hemorrhoidal Disease | Bipolar disorder | rs6867042 | T | C | -0.035 | 6.02E-09 | 0.006 | 33.83 |
| Hemorrhoidal Disease | Bipolar disorder | rs72707023 | A | G | -0.039 | 2.37E-13 | 0.005 | 53.32 |
| Hemorrhoidal Disease | Bipolar disorder | rs728327 | C | T | 0.026 | 2.98E-10 | 0.004 | 40.52 |
| Hemorrhoidal Disease | Bipolar disorder | rs755209 | A | G | 0.024 | 3.06E-08 | 0.004 | 30.50 |
| Hemorrhoidal Disease | Bipolar disorder | rs7594056 | G | A | 0.035 | 7.22E-15 | 0.004 | 61.48 |
| Hemorrhoidal Disease | Bipolar disorder | rs7749659 | G | A | -0.027 | 2.91E-09 | 0.005 | 34.71 |
| Hemorrhoidal Disease | Bipolar disorder | rs7795564 | A | G | -0.029 | 3.38E-13 | 0.004 | 54.02 |
| Hemorrhoidal Disease | Bipolar disorder | rs78378222 | G | T | 0.112 | 4.66E-10 | 0.018 | 38.79 |
| Hemorrhoidal Disease | Bipolar disorder | rs7994724 | G | A | -0.035 | 1.75E-17 | 0.004 | 71.63 |
| Hemorrhoidal Disease | Bipolar disorder | rs8106090 | G | A | 0.022 | 3.34E-08 | 0.004 | 30.96 |
| Hemorrhoidal Disease | Bipolar disorder | rs900400 | C | T | -0.035 | 5.02E-18 | 0.004 | 74.82 |
| Hemorrhoidal Disease | Bipolar disorder | rs9306894 | G | A | -0.026 | 2.34E-10 | 0.004 | 39.91 |
| Hemorrhoidal Disease | Bipolar disorder | rs9322356 | A | G | 0.040 | 2.01E-08 | 0.007 | 31.74 |
| Hemorrhoidal Disease | Bipolar disorder | rs9847710 | C | T | 0.040 | 1.23E-23 | 0.004 | 99.50 |
| Hemorrhoidal Disease | Bipolar disorder | rs9853475 | A | G | 0.026 | 1.96E-09 | 0.004 | 35.73 |
| Hemorrhoidal Disease | Depression | rs10153716 | G | A | 0.024 | 9.10E-09 | 0.004 | 32.65 |
| Hemorrhoidal Disease | Depression | rs10807610 | C | A | 0.037 | 2.30E-12 | 0.005 | 49.26 |
| Hemorrhoidal Disease | Depression | rs11045079 | G | A | -0.035 | 1.79E-09 | 0.006 | 36.83 |
| Hemorrhoidal Disease | Depression | rs11176001 | A | C | -0.084 | 2.48E-46 | 0.006 | 203.18 |
| Hemorrhoidal Disease | Depression | rs1156533 | G | A | -0.024 | 1.76E-08 | 0.004 | 31.94 |
| Hemorrhoidal Disease | Depression | rs11635984 | C | T | -0.029 | 4.14E-13 | 0.004 | 53.66 |
| Hemorrhoidal Disease | Depression | rs11770437 | T | C | -0.038 | 3.18E-18 | 0.004 | 74.19 |
| Hemorrhoidal Disease | Depression | rs11942410 | T | C | -0.027 | 1.14E-08 | 0.005 | 32.27 |
| Hemorrhoidal Disease | Depression | rs12654883 | T | C | 0.025 | 1.19E-08 | 0.004 | 32.03 |
| Hemorrhoidal Disease | Depression | rs13632 | G | A | -0.030 | 6.12E-11 | 0.005 | 43.67 |
| Hemorrhoidal Disease | Depression | rs1542726 | C | A | -0.028 | 2.53E-12 | 0.004 | 49.70 |
| Hemorrhoidal Disease | Depression | rs16831319 | C | T | 0.048 | 9.95E-09 | 0.008 | 32.52 |
| Hemorrhoidal Disease | Depression | rs1689549 | C | T | -0.038 | 4.43E-09 | 0.006 | 34.33 |
| Hemorrhoidal Disease | Depression | rs17293632 | T | C | 0.054 | 6.88E-32 | 0.005 | 139.34 |
| Hemorrhoidal Disease | Depression | rs174767 | G | A | 0.023 | 1.45E-08 | 0.004 | 31.47 |
| Hemorrhoidal Disease | Depression | rs17824374 | C | T | -0.033 | 3.35E-11 | 0.005 | 44.54 |
| Hemorrhoidal Disease | Depression | rs1838392 | G | T | -0.053 | 4.60E-39 | 0.004 | 172.27 |
| Hemorrhoidal Disease | Depression | rs1858015 | T | C | 0.028 | 6.09E-11 | 0.004 | 43.18 |
| Hemorrhoidal Disease | Depression | rs2060285 | A | C | 0.025 | 4.19E-09 | 0.004 | 34.03 |
| Hemorrhoidal Disease | Depression | rs2186797 | C | T | -0.044 | 4.46E-08 | 0.008 | 29.64 |
| Hemorrhoidal Disease | Depression | rs2327426 | C | T | -0.026 | 2.68E-09 | 0.004 | 35.72 |
| Hemorrhoidal Disease | Depression | rs2525570 | G | A | 0.025 | 6.79E-10 | 0.004 | 39.06 |
| Hemorrhoidal Disease | Depression | rs2555004 | A | G | 0.025 | 5.36E-10 | 0.004 | 39.46 |
| Hemorrhoidal Disease | Depression | rs2605097 | A | C | -0.025 | 4.27E-09 | 0.004 | 34.07 |
| Hemorrhoidal Disease | Depression | rs2687965 | A | G | -0.023 | 5.91E-09 | 0.004 | 33.64 |
| Hemorrhoidal Disease | Depression | rs2832279 | A | C | 0.027 | 4.46E-11 | 0.004 | 44.01 |
| Hemorrhoidal Disease | Depression | rs2861709 | G | A | 0.051 | 2.38E-09 | 0.009 | 35.30 |
| Hemorrhoidal Disease | Depression | rs3012065 | C | T | 0.024 | 4.13E-08 | 0.004 | 30.38 |
| Hemorrhoidal Disease | Depression | rs3253 | T | C | 0.029 | 4.96E-12 | 0.004 | 48.01 |
| Hemorrhoidal Disease | Depression | rs34161672 | A | G | -0.030 | 4.18E-12 | 0.004 | 47.42 |
| Hemorrhoidal Disease | Depression | rs3757582 | C | T | 0.056 | 1.69E-10 | 0.009 | 41.14 |
| Hemorrhoidal Disease | Depression | rs4233681 | C | T | -0.026 | 2.04E-11 | 0.004 | 45.82 |
| Hemorrhoidal Disease | Depression | rs4345978 | T | G | -0.034 | 1.20E-13 | 0.005 | 54.95 |
| Hemorrhoidal Disease | Depression | rs4423457 | A | G | -0.022 | 3.36E-08 | 0.004 | 30.53 |
| Hemorrhoidal Disease | Depression | rs4556017 | T | C | -0.057 | 1.30E-22 | 0.006 | 96.24 |
| Hemorrhoidal Disease | Depression | rs4579999 | C | T | -0.029 | 3.32E-13 | 0.004 | 52.20 |
| Hemorrhoidal Disease | Depression | rs4679900 | T | C | 0.023 | 9.52E-09 | 0.004 | 33.06 |
| Hemorrhoidal Disease | Depression | rs4843407 | A | G | -0.031 | 4.03E-12 | 0.005 | 47.15 |
| Hemorrhoidal Disease | Depression | rs4951080 | G | A | 0.026 | 2.81E-09 | 0.004 | 35.17 |
| Hemorrhoidal Disease | Depression | rs56269620 | G | A | 0.034 | 2.31E-16 | 0.004 | 66.76 |
| Hemorrhoidal Disease | Depression | rs57116599 | A | G | 0.032 | 1.48E-11 | 0.005 | 46.07 |
| Hemorrhoidal Disease | Depression | rs58579887 | C | T | 0.028 | 1.67E-11 | 0.004 | 44.76 |
| Hemorrhoidal Disease | Depression | rs61026653 | G | A | -0.033 | 7.62E-09 | 0.006 | 32.91 |
| Hemorrhoidal Disease | Depression | rs62061554 | A | G | 0.039 | 2.64E-10 | 0.006 | 39.98 |
| Hemorrhoidal Disease | Depression | rs62368263 | C | T | -0.044 | 5.91E-15 | 0.006 | 62.02 |
| Hemorrhoidal Disease | Depression | rs6462976 | C | T | 0.028 | 1.23E-12 | 0.004 | 51.55 |
| Hemorrhoidal Disease | Depression | rs6482359 | G | A | -0.027 | 3.14E-11 | 0.004 | 43.37 |
| Hemorrhoidal Disease | Depression | rs6498573 | T | C | 0.042 | 5.20E-12 | 0.006 | 48.30 |
| Hemorrhoidal Disease | Depression | rs6723226 | G | A | 0.024 | 1.99E-08 | 0.004 | 31.57 |
| Hemorrhoidal Disease | Depression | rs6792493 | G | A | 0.031 | 9.52E-15 | 0.004 | 59.68 |
| Hemorrhoidal Disease | Depression | rs6839705 | C | A | -0.028 | 5.05E-12 | 0.004 | 46.97 |
| Hemorrhoidal Disease | Depression | rs6867042 | T | C | -0.035 | 6.02E-09 | 0.006 | 33.83 |
| Hemorrhoidal Disease | Depression | rs7183672 | G | A | 0.031 | 1.20E-13 | 0.004 | 55.54 |
| Hemorrhoidal Disease | Depression | rs72707023 | A | G | -0.039 | 2.37E-13 | 0.005 | 53.32 |
| Hemorrhoidal Disease | Depression | rs755209 | A | G | 0.024 | 3.06E-08 | 0.004 | 30.50 |
| Hemorrhoidal Disease | Depression | rs7594056 | G | A | 0.035 | 7.22E-15 | 0.004 | 61.48 |
| Hemorrhoidal Disease | Depression | rs7749659 | G | A | -0.027 | 2.91E-09 | 0.005 | 34.71 |
| Hemorrhoidal Disease | Depression | rs7795564 | A | G | -0.029 | 3.38E-13 | 0.004 | 54.02 |
| Hemorrhoidal Disease | Depression | rs8106090 | G | A | 0.022 | 3.34E-08 | 0.004 | 30.96 |
| Hemorrhoidal Disease | Depression | rs9306894 | G | A | -0.026 | 2.34E-10 | 0.004 | 39.91 |
| Hemorrhoidal Disease | Depression | rs9322356 | A | G | 0.040 | 2.01E-08 | 0.007 | 31.74 |
| Hemorrhoidal Disease | Depression | rs9847710 | C | T | 0.040 | 1.23E-23 | 0.004 | 99.50 |
| Hemorrhoidal Disease | Depression | rs9853475 | A | G | 0.026 | 1.96E-09 | 0.004 | 35.73 |
| Hemorrhoidal Disease | Schizophrenia | rs10153716 | G | A | 0.024 | 9.10E-09 | 0.004 | 32.65 |
| Hemorrhoidal Disease | Schizophrenia | rs10807610 | C | A | 0.037 | 2.30E-12 | 0.005 | 49.26 |
| Hemorrhoidal Disease | Schizophrenia | rs10838738 | G | A | 0.025 | 1.69E-09 | 0.004 | 36.59 |
| Hemorrhoidal Disease | Schizophrenia | rs10956488 | G | A | 0.033 | 1.66E-09 | 0.006 | 36.22 |
| Hemorrhoidal Disease | Schizophrenia | rs11045079 | G | A | -0.035 | 1.79E-09 | 0.006 | 36.83 |
| Hemorrhoidal Disease | Schizophrenia | rs11176001 | A | C | -0.084 | 2.48E-46 | 0.006 | 203.18 |
| Hemorrhoidal Disease | Schizophrenia | rs1156533 | G | A | -0.024 | 1.76E-08 | 0.004 | 31.94 |
| Hemorrhoidal Disease | Schizophrenia | rs11578225 | A | G | -0.034 | 6.36E-13 | 0.005 | 51.36 |
| Hemorrhoidal Disease | Schizophrenia | rs11635984 | C | T | -0.029 | 4.14E-13 | 0.004 | 53.66 |
| Hemorrhoidal Disease | Schizophrenia | rs11770437 | T | C | -0.038 | 3.18E-18 | 0.004 | 74.19 |
| Hemorrhoidal Disease | Schizophrenia | rs11942410 | T | C | -0.027 | 1.14E-08 | 0.005 | 32.27 |
| Hemorrhoidal Disease | Schizophrenia | rs12153515 | T | C | 0.037 | 7.33E-10 | 0.006 | 38.48 |
| Hemorrhoidal Disease | Schizophrenia | rs12654883 | T | C | 0.025 | 1.19E-08 | 0.004 | 32.03 |
| Hemorrhoidal Disease | Schizophrenia | rs13632 | G | A | -0.030 | 6.12E-11 | 0.005 | 43.67 |
| Hemorrhoidal Disease | Schizophrenia | rs145163454 | C | T | -0.143 | 5.88E-28 | 0.013 | 120.66 |
| Hemorrhoidal Disease | Schizophrenia | rs1542726 | C | A | -0.028 | 2.53E-12 | 0.004 | 49.70 |
| Hemorrhoidal Disease | Schizophrenia | rs16831319 | C | T | 0.048 | 9.95E-09 | 0.008 | 32.52 |
| Hemorrhoidal Disease | Schizophrenia | rs1689549 | C | T | -0.038 | 4.43E-09 | 0.006 | 34.33 |
| Hemorrhoidal Disease | Schizophrenia | rs17105613 | C | T | -0.025 | 2.67E-09 | 0.004 | 35.43 |
| Hemorrhoidal Disease | Schizophrenia | rs17293632 | T | C | 0.054 | 6.88E-32 | 0.005 | 139.34 |
| Hemorrhoidal Disease | Schizophrenia | rs174767 | G | A | 0.023 | 1.45E-08 | 0.004 | 31.47 |
| Hemorrhoidal Disease | Schizophrenia | rs17824374 | C | T | -0.033 | 3.35E-11 | 0.005 | 44.54 |
| Hemorrhoidal Disease | Schizophrenia | rs1838392 | G | T | -0.053 | 4.60E-39 | 0.004 | 172.27 |
| Hemorrhoidal Disease | Schizophrenia | rs1858015 | T | C | 0.028 | 6.09E-11 | 0.004 | 43.18 |
| Hemorrhoidal Disease | Schizophrenia | rs2060285 | A | C | 0.025 | 4.19E-09 | 0.004 | 34.03 |
| Hemorrhoidal Disease | Schizophrenia | rs2186797 | C | T | -0.044 | 4.46E-08 | 0.008 | 29.64 |
| Hemorrhoidal Disease | Schizophrenia | rs2212450 | C | T | 0.027 | 5.43E-11 | 0.004 | 43.37 |
| Hemorrhoidal Disease | Schizophrenia | rs2327426 | C | T | -0.026 | 2.68E-09 | 0.004 | 35.72 |
| Hemorrhoidal Disease | Schizophrenia | rs2421206 | G | T | 0.033 | 2.29E-14 | 0.004 | 58.90 |
| Hemorrhoidal Disease | Schizophrenia | rs2525570 | G | A | 0.025 | 6.79E-10 | 0.004 | 39.06 |
| Hemorrhoidal Disease | Schizophrenia | rs2555004 | A | G | 0.025 | 5.36E-10 | 0.004 | 39.46 |
| Hemorrhoidal Disease | Schizophrenia | rs2605097 | A | C | -0.025 | 4.27E-09 | 0.004 | 34.07 |
| Hemorrhoidal Disease | Schizophrenia | rs2687965 | A | G | -0.023 | 5.91E-09 | 0.004 | 33.64 |
| Hemorrhoidal Disease | Schizophrenia | rs2832279 | A | C | 0.027 | 4.46E-11 | 0.004 | 44.01 |
| Hemorrhoidal Disease | Schizophrenia | rs28663472 | T | C | -0.025 | 4.69E-10 | 0.004 | 39.46 |
| Hemorrhoidal Disease | Schizophrenia | rs3012065 | C | T | 0.024 | 4.13E-08 | 0.004 | 30.38 |
| Hemorrhoidal Disease | Schizophrenia | rs3253 | T | C | 0.029 | 4.96E-12 | 0.004 | 48.01 |
| Hemorrhoidal Disease | Schizophrenia | rs34161672 | A | G | -0.030 | 4.18E-12 | 0.004 | 47.42 |
| Hemorrhoidal Disease | Schizophrenia | rs3757582 | C | T | 0.056 | 1.69E-10 | 0.009 | 41.14 |
| Hemorrhoidal Disease | Schizophrenia | rs4233681 | C | T | -0.026 | 2.04E-11 | 0.004 | 45.82 |
| Hemorrhoidal Disease | Schizophrenia | rs4345978 | T | G | -0.034 | 1.20E-13 | 0.005 | 54.95 |
| Hemorrhoidal Disease | Schizophrenia | rs4423457 | A | G | -0.022 | 3.36E-08 | 0.004 | 30.53 |
| Hemorrhoidal Disease | Schizophrenia | rs4556017 | T | C | -0.057 | 1.30E-22 | 0.006 | 96.24 |
| Hemorrhoidal Disease | Schizophrenia | rs4579999 | C | T | -0.029 | 3.32E-13 | 0.004 | 52.20 |
| Hemorrhoidal Disease | Schizophrenia | rs4679900 | T | C | 0.023 | 9.52E-09 | 0.004 | 33.06 |
| Hemorrhoidal Disease | Schizophrenia | rs4843407 | A | G | -0.031 | 4.03E-12 | 0.005 | 47.15 |
| Hemorrhoidal Disease | Schizophrenia | rs4951080 | G | A | 0.026 | 2.81E-09 | 0.004 | 35.17 |
| Hemorrhoidal Disease | Schizophrenia | rs56269620 | G | A | 0.034 | 2.31E-16 | 0.004 | 66.76 |
| Hemorrhoidal Disease | Schizophrenia | rs57116599 | A | G | 0.032 | 1.48E-11 | 0.005 | 46.07 |
| Hemorrhoidal Disease | Schizophrenia | rs58579887 | C | T | 0.028 | 1.67E-11 | 0.004 | 44.76 |
| Hemorrhoidal Disease | Schizophrenia | rs61026653 | G | A | -0.033 | 7.62E-09 | 0.006 | 32.91 |
| Hemorrhoidal Disease | Schizophrenia | rs62061554 | A | G | 0.039 | 2.64E-10 | 0.006 | 39.98 |
| Hemorrhoidal Disease | Schizophrenia | rs62368263 | C | T | -0.044 | 5.91E-15 | 0.006 | 62.02 |
| Hemorrhoidal Disease | Schizophrenia | rs6462976 | C | T | 0.028 | 1.23E-12 | 0.004 | 51.55 |
| Hemorrhoidal Disease | Schizophrenia | rs6482359 | G | A | -0.027 | 3.14E-11 | 0.004 | 43.37 |
| Hemorrhoidal Disease | Schizophrenia | rs6498573 | T | C | 0.042 | 5.20E-12 | 0.006 | 48.30 |
| Hemorrhoidal Disease | Schizophrenia | rs6723226 | G | A | 0.024 | 1.99E-08 | 0.004 | 31.57 |
| Hemorrhoidal Disease | Schizophrenia | rs6792493 | G | A | 0.031 | 9.52E-15 | 0.004 | 59.68 |
| Hemorrhoidal Disease | Schizophrenia | rs6839705 | C | A | -0.028 | 5.05E-12 | 0.004 | 46.97 |
| Hemorrhoidal Disease | Schizophrenia | rs6867042 | T | C | -0.035 | 6.02E-09 | 0.006 | 33.83 |
| Hemorrhoidal Disease | Schizophrenia | rs7183672 | G | A | 0.031 | 1.20E-13 | 0.004 | 55.54 |
| Hemorrhoidal Disease | Schizophrenia | rs72707023 | A | G | -0.039 | 2.37E-13 | 0.005 | 53.32 |
| Hemorrhoidal Disease | Schizophrenia | rs728327 | C | T | 0.026 | 2.98E-10 | 0.004 | 40.52 |
| Hemorrhoidal Disease | Schizophrenia | rs755209 | A | G | 0.024 | 3.06E-08 | 0.004 | 30.50 |
| Hemorrhoidal Disease | Schizophrenia | rs7594056 | G | A | 0.035 | 7.22E-15 | 0.004 | 61.48 |
| Hemorrhoidal Disease | Schizophrenia | rs7749659 | G | A | -0.027 | 2.91E-09 | 0.005 | 34.71 |
| Hemorrhoidal Disease | Schizophrenia | rs7795564 | A | G | -0.029 | 3.38E-13 | 0.004 | 54.02 |
| Hemorrhoidal Disease | Schizophrenia | rs78378222 | G | T | 0.112 | 4.66E-10 | 0.018 | 38.79 |
| Hemorrhoidal Disease | Schizophrenia | rs7994724 | G | A | -0.035 | 1.75E-17 | 0.004 | 71.63 |
| Hemorrhoidal Disease | Schizophrenia | rs8106090 | G | A | 0.022 | 3.34E-08 | 0.004 | 30.96 |
| Hemorrhoidal Disease | Schizophrenia | rs900400 | C | T | -0.035 | 5.02E-18 | 0.004 | 74.82 |
| Hemorrhoidal Disease | Schizophrenia | rs9306894 | G | A | -0.026 | 2.34E-10 | 0.004 | 39.91 |
| Hemorrhoidal Disease | Schizophrenia | rs9322356 | A | G | 0.040 | 2.01E-08 | 0.007 | 31.74 |
| Hemorrhoidal Disease | Schizophrenia | rs9847710 | C | T | 0.040 | 1.23E-23 | 0.004 | 99.50 |
| Hemorrhoidal Disease | Schizophrenia | rs9853475 | A | G | 0.026 | 1.96E-09 | 0.004 | 35.73 |

Remark:SNP: single nucleotide polymorphism; SE: standard error. *β* stands for the estimated coefficient of exposures via extracting their SNPs in outcome genetic information after screening LD.

**Supplementary Table 2**:The result of MR-PRESSO between Depression, Bipolar disorder, Anxiety disorders, Schizophrenia, and Hemorrhoidal Disease.

| Exposure | Outcome | MR Analysis | Estimate | Sd | T-stat | P-value | Global.Test.RSSobs | Global.Test.Pvalue |
| --- | --- | --- | --- | --- | --- | --- | --- | --- |
| Anxiety disorders | Hemorrhoidal Disease | Raw | 0.139 | 0.059 | 2.338 | 0.048 | 32.192 | 0.010 |
| Anxiety disorders | Hemorrhoidal Disease | Outlier-corrected | 0.034 | 0.022 | 1.556 | 0.171 | 32.192 | 0.010 |
| Bipolar disorder | Hemorrhoidal Disease | Raw | 0.033 | 0.021 | 1.526 | 0.134 | 193.616 | <0.001 |
| Bipolar disorder | Hemorrhoidal Disease | Outlier-corrected | 0.024 | 0.015 | 1.581 | 0.122 | 193.616 | <0.001 |
| Depression | Hemorrhoidal Disease | Raw | 0.170 | 0.050 | 3.290 | <0.01 | 77.630 | <0.001 |
| Depression | Hemorrhoidal Disease | Outlier-corrected | 0.190 | 0.050 | 3.850 | <0.01 | 77.630 | <0.001 |
| Schizophrenia | Hemorrhoidal Disease | Raw | -0.007 | 0.006 | -1.178 | 0.25 | 24.926 | 0.636 |
| Schizophrenia | Hemorrhoidal Disease | Outlier-corrected | NA | NA | NA | NA | 24.926 | 0.636 |
| Hemorrhoidal Disease | Anxiety disorders | Raw | 0.050 | 0.039 | 1.282 | 0.204 | 147.130 | <0.001 |
| Hemorrhoidal Disease | Anxiety disorders | Outlier-corrected | 0.049 | 0.036 | 1.347 | 0.182 | 147.130 | <0.001 |
| Hemorrhoidal Disease | Bipolar disorder | Raw | 0.080 | 0.059 | 1.348 | 0.182 | 181.703 | <0.001 |
| Hemorrhoidal Disease | Bipolar disorder | Outlier-corrected | 0.028 | 0.052 | 0.535 | 0.595 | 181.703 | <0.001 |
| Hemorrhoidal Disease | Depression | Raw | 0.061 | 0.019 | 3.167 | 0.002 | 87.859 | 0.038 |
| Hemorrhoidal Disease | Depression | Outlier-corrected | 0.070 | 0.018 | 3.929 | <0.001 | 87.859 | 0.038 |
| Hemorrhoidal Disease | Schizophrenia | Raw | -0.016 | 0.118 | -0.136 | 0.892 | 118.615 | 0.008 |
| Hemorrhoidal Disease | Schizophrenia | Outlier-corrected | NA | NA | NA | NA | 118.615 | 0.008 |

Remark: RSS represents the residual sum of squares calculated by the MR-PRESSO Global Test. Causal estimation means the estimated coefficient from the MR-PRESSO outlier test. If P-value > 0.05 means non-significant sensitivity from the outlier test, then the result of Outlier-corrected displays "NA" in R software.

**Supplementary Figures**


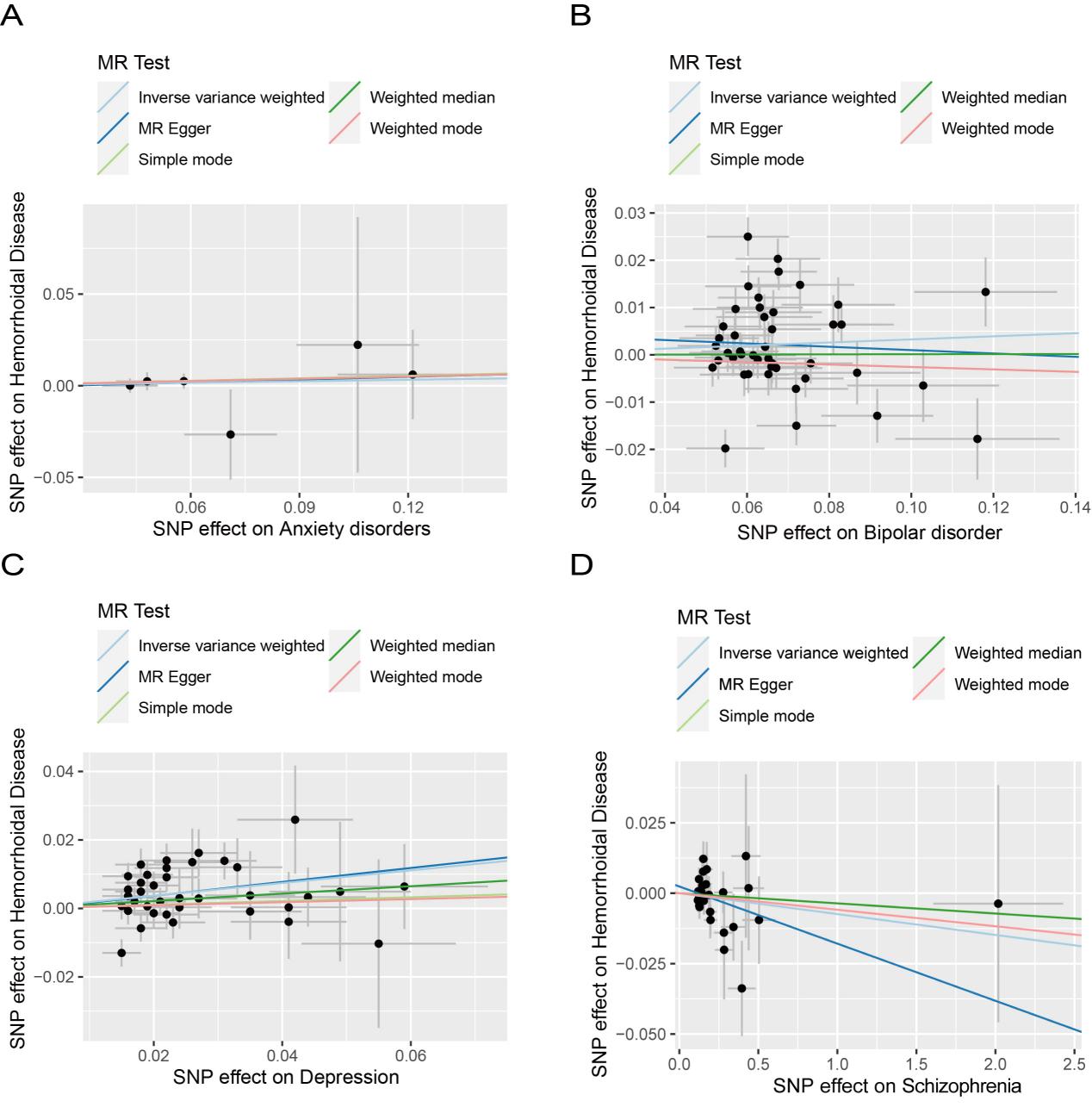


**Figure S1** Scatter plot of the effect size of each single-nucleotide polymorphism on Anxiety disorders (A), Bipolar disorder (B), Depression (C), and Schizophrenia (D), and Hemorrhoidal Disease risk using Mendelian randomization approaches, including inverse variance-weighted, weighted median, MR-Egger, weighted mode and simple mode respectively.


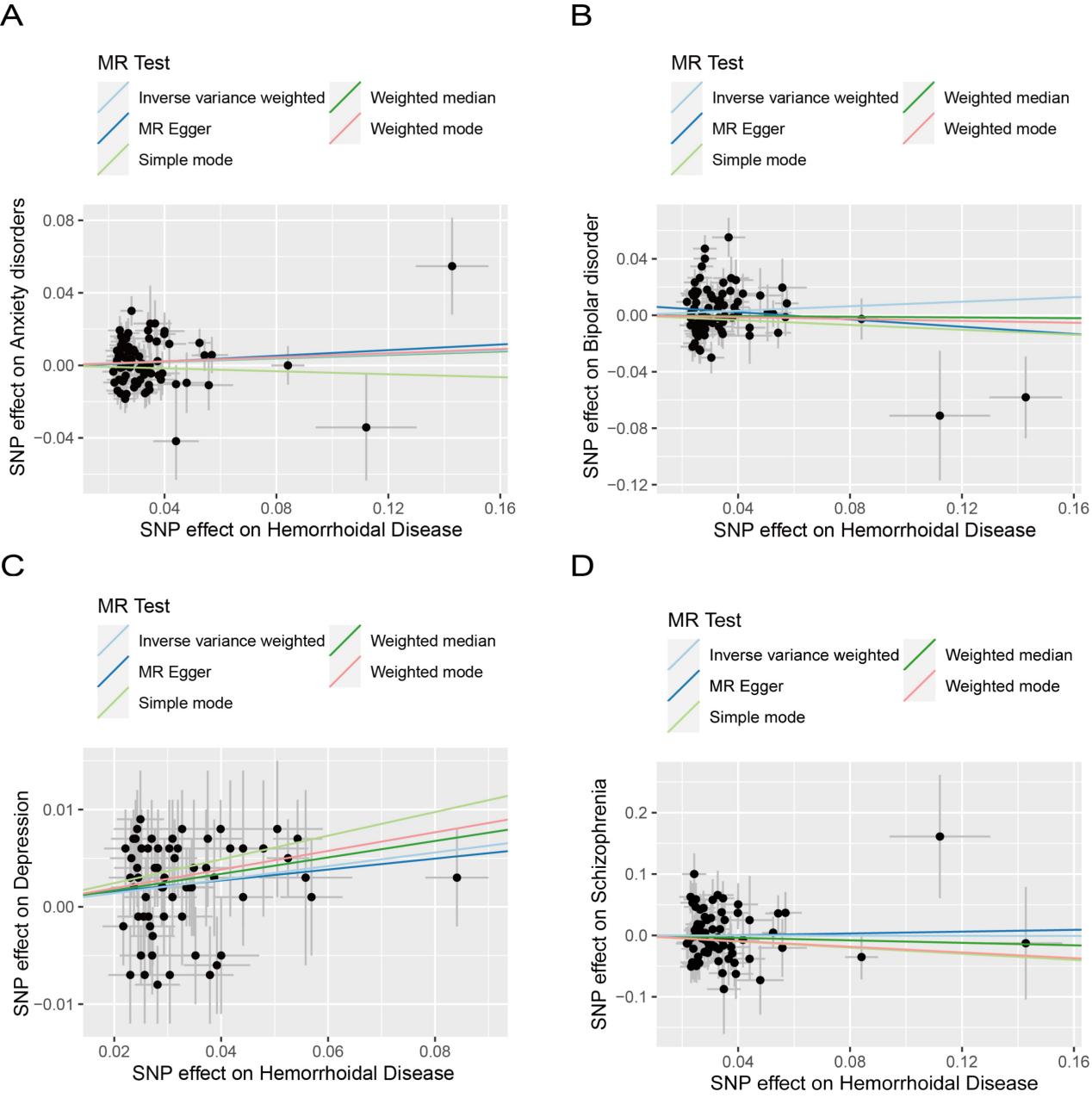


**Figure S2** Scatter plot of the effect size of each single-nucleotide polymorphism on Hemorrhoidal Disease and Anxiety disorders risk(A), Bipolar disorder risk(B), Depression risk(C), and Schizophrenia risk(D), using Mendelian randomization approaches, including inverse variance-weighted, weighted median, MR-Egger, weighted mode and simple mode respectively.


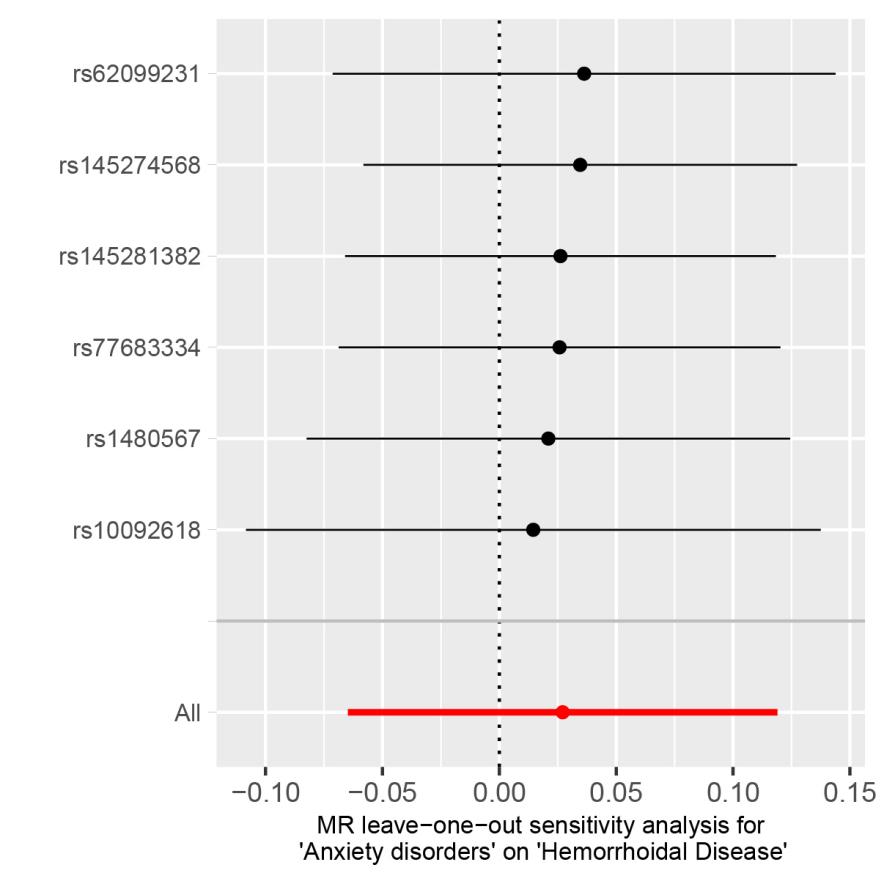


**Figure S3.** Leave-one-out analysis of the causal effect of Anxiety disorders on Hemorrhoidal Disease


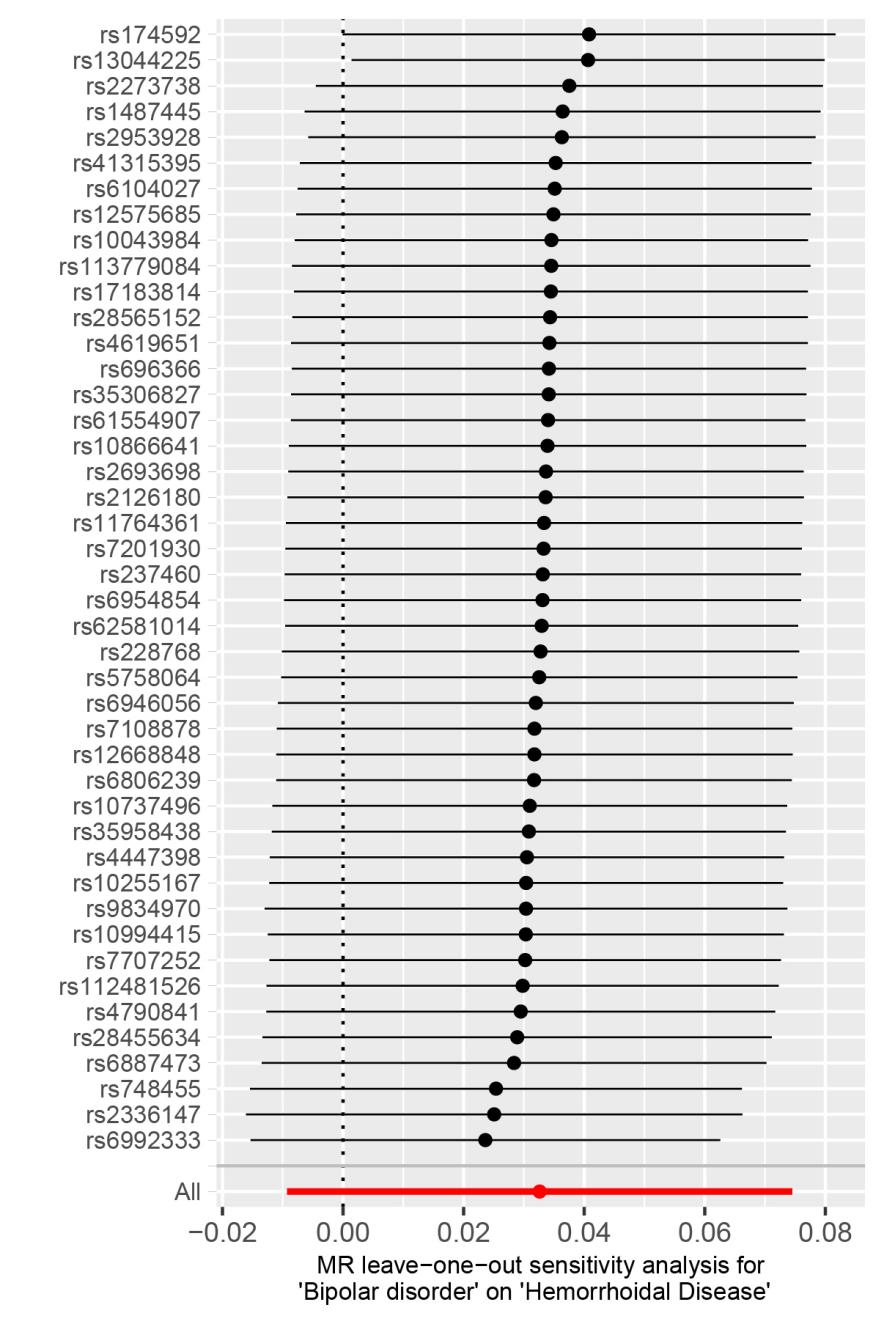


**Figure S4.** Leave-one-out analysis of the causal effect of Bipolar disorder on Hemorrhoidal Disease


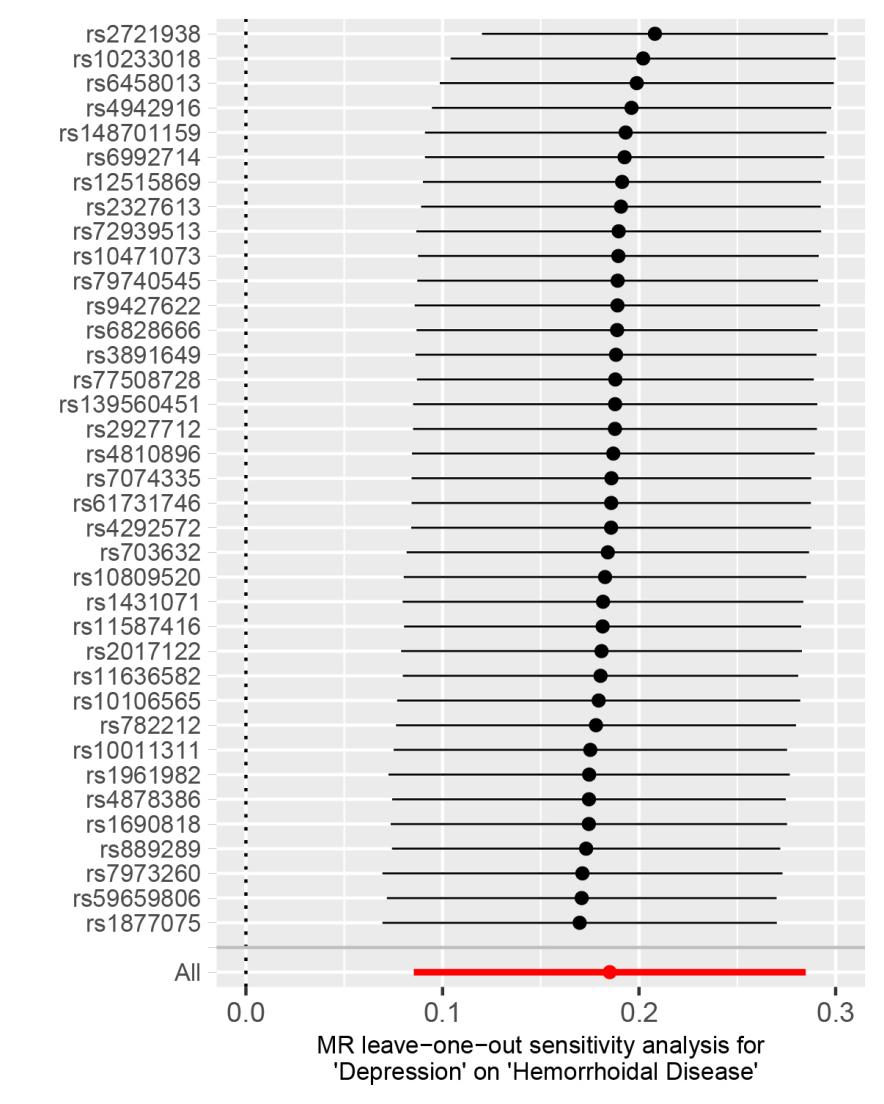


**Figure S5.** Leave-one-out analysis of the causal effect of Depression on Hemorrhoidal Disease


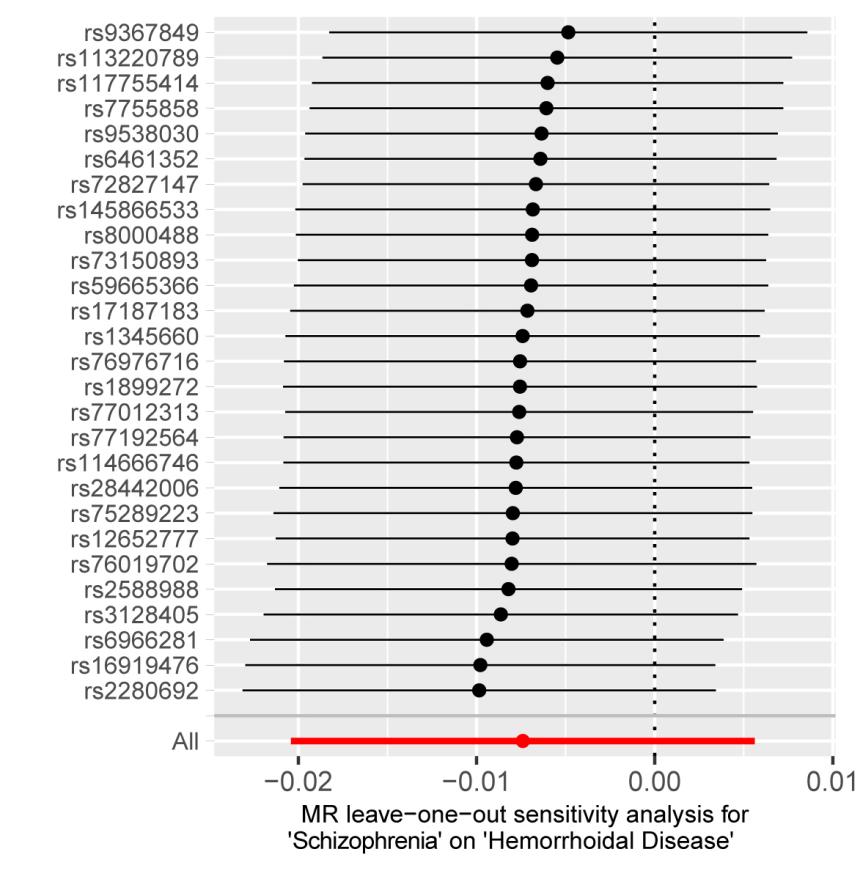


**Figure S6.** Leave-one-out analysis of the causal effect of Schizophrenia on Hemorrhoidal Disease


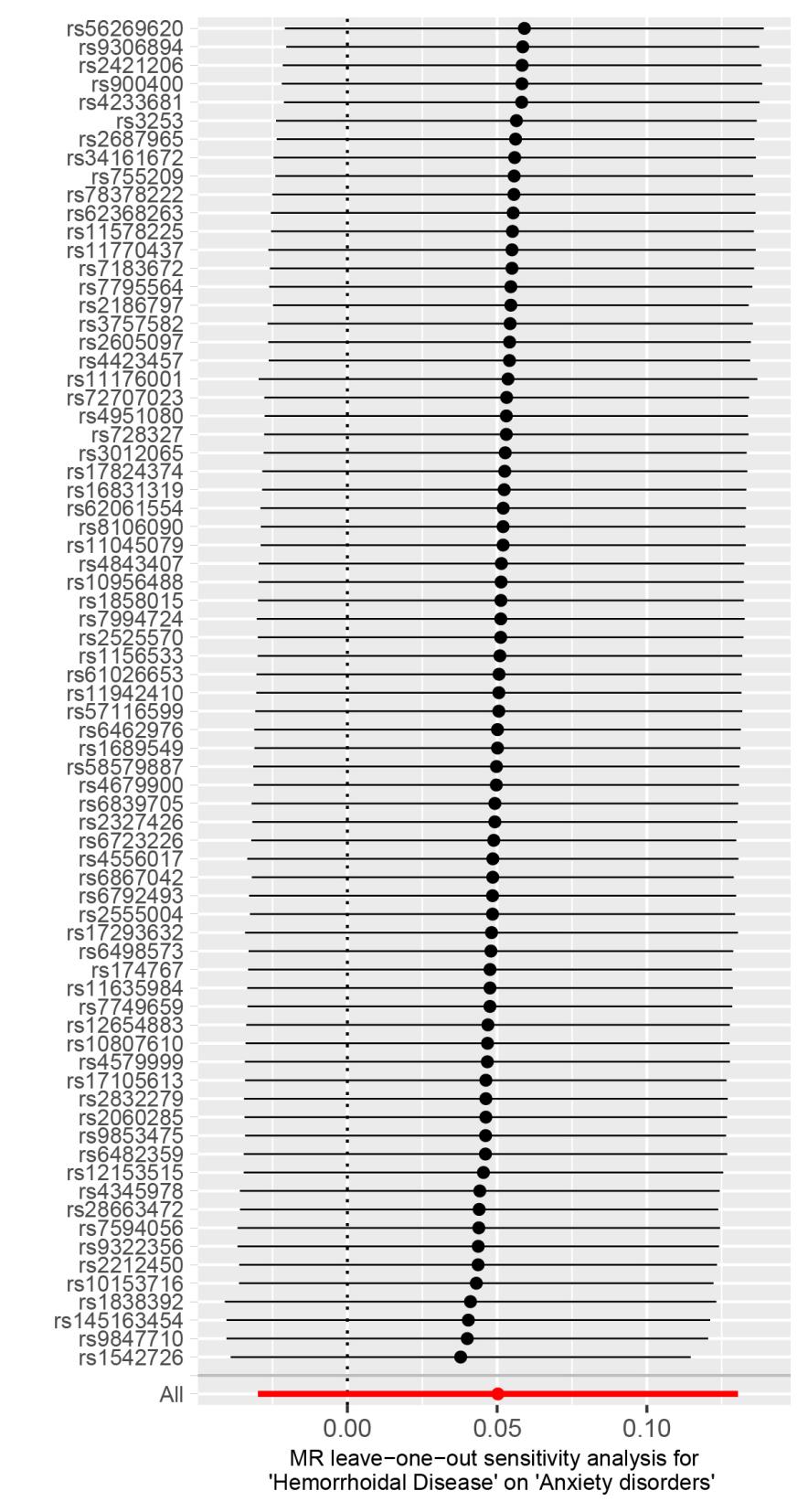


**Figure S7.** Leave-one-out analysis of the causal effect of Hemorrhoidal Disease on Anxiety disorders


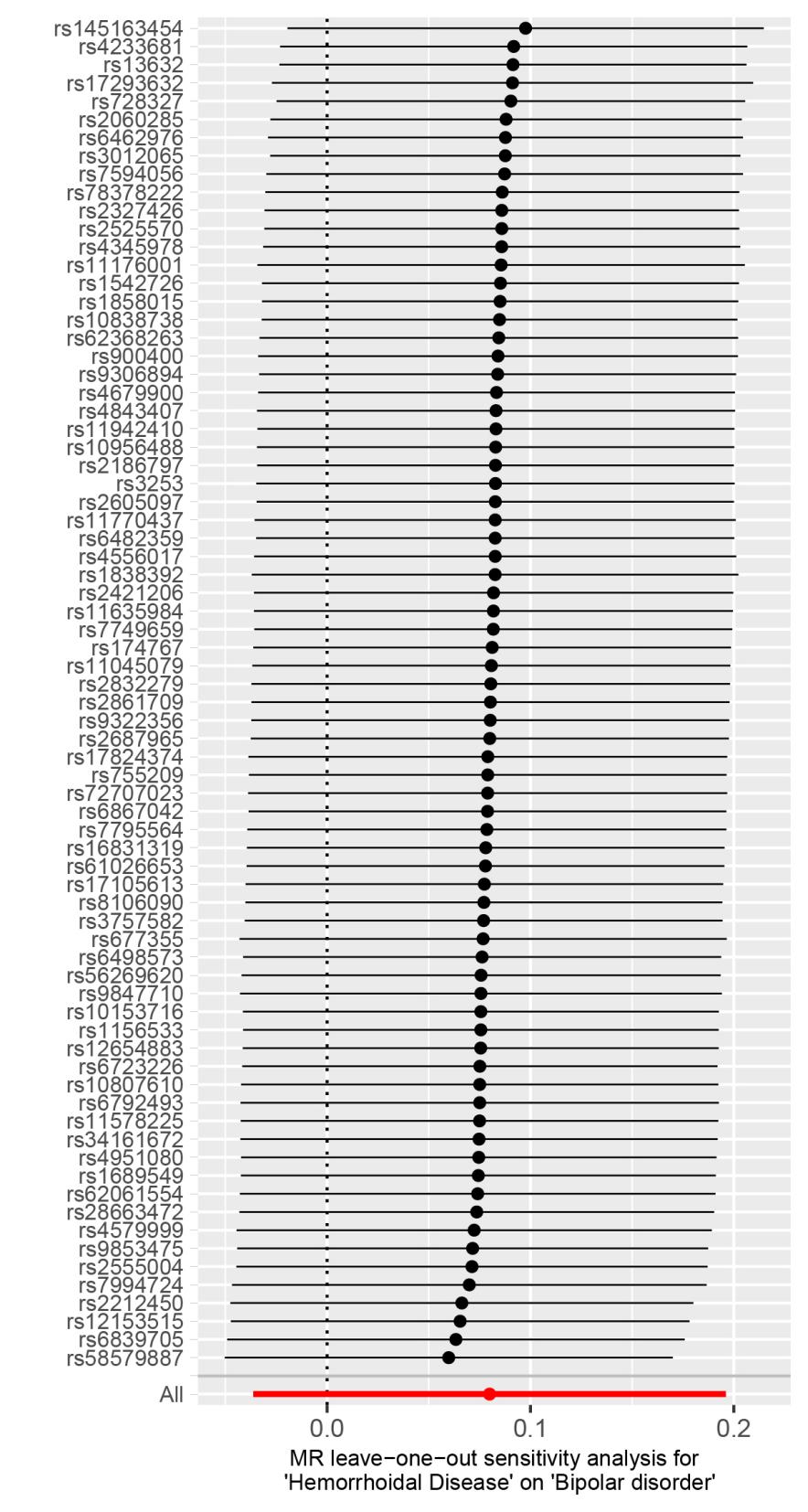


**Figure S8.** Leave-one-out analysis of the causal effect of Hemorrhoidal Disease on Bipolar disorder


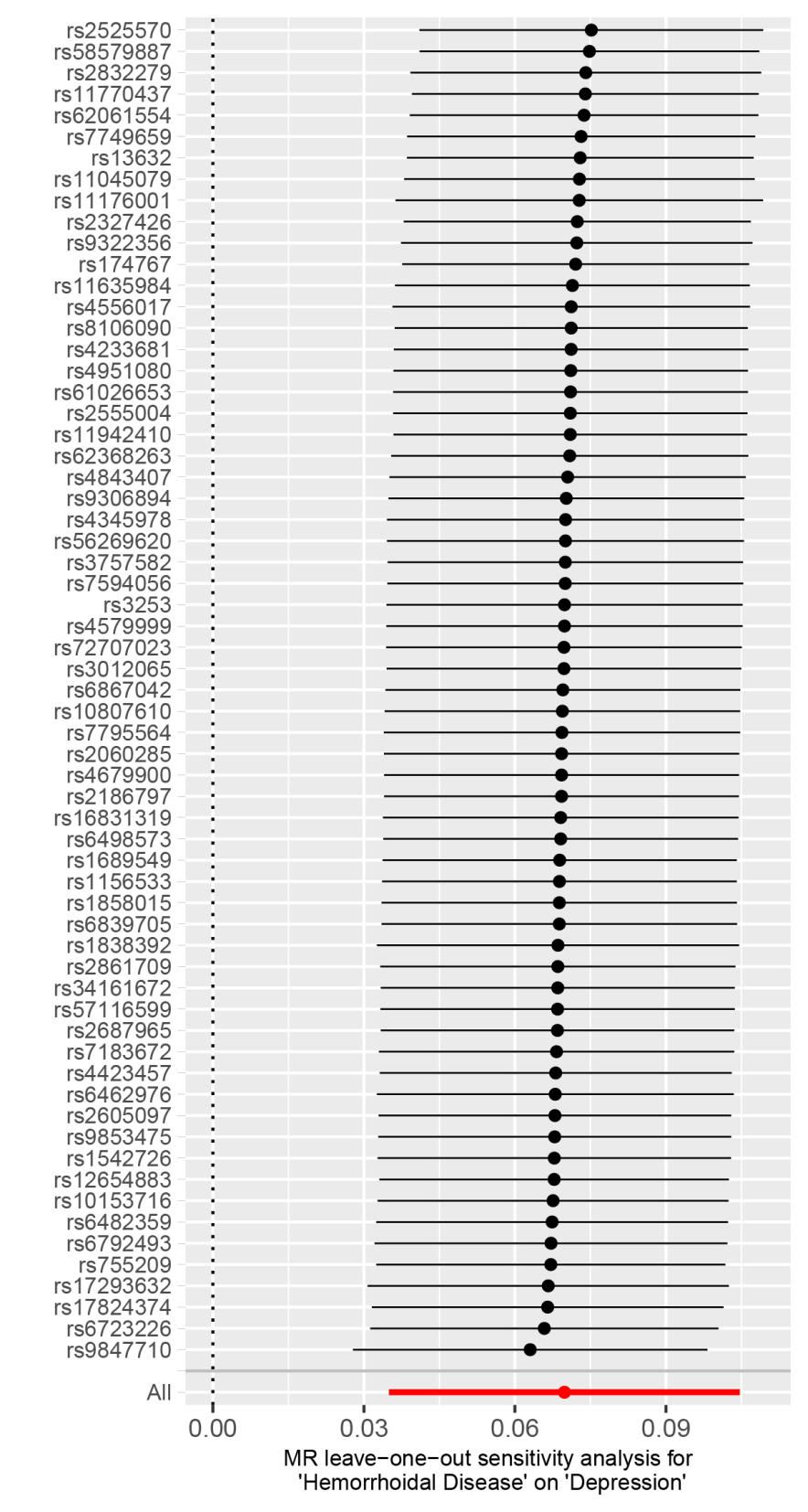


**Figure S9.** Leave-one-out analysis of the causal effect of Hemorrhoidal Disease on Depression


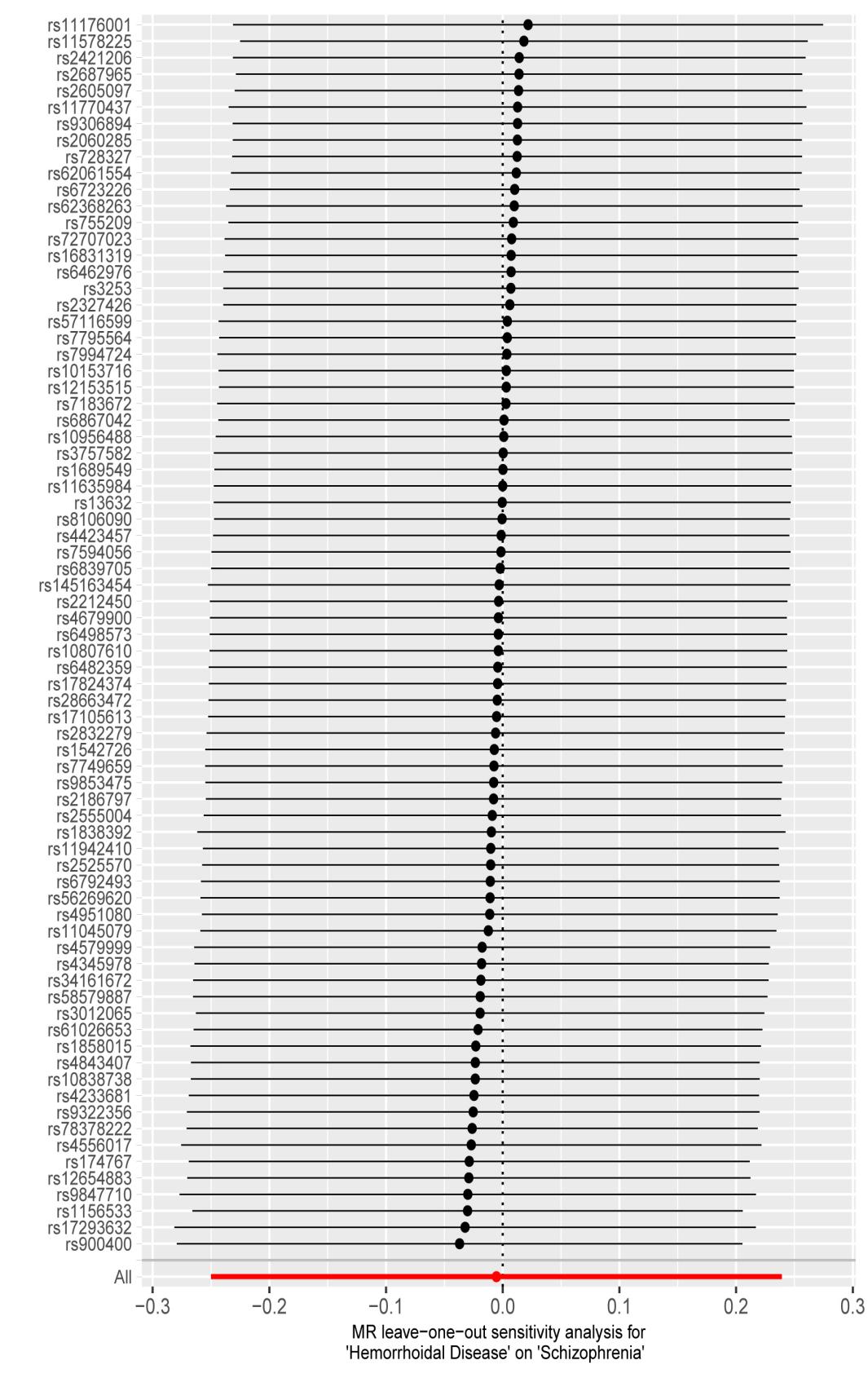


**Figure S10.** Leave-one-out analysis of the causal effect of Hemorrhoidal Disease on Schizophrenia


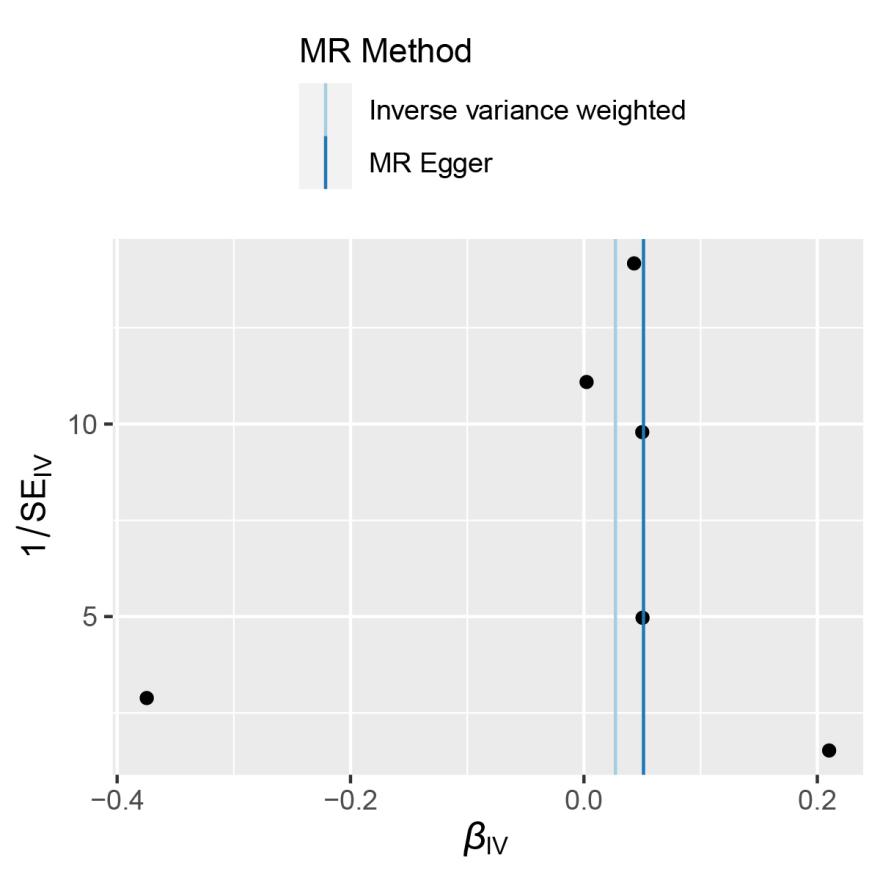


**Figure S11.** Funnel plot for MR analyses of causal effect of Anxiety disorders on Hemorrhoidal Disease


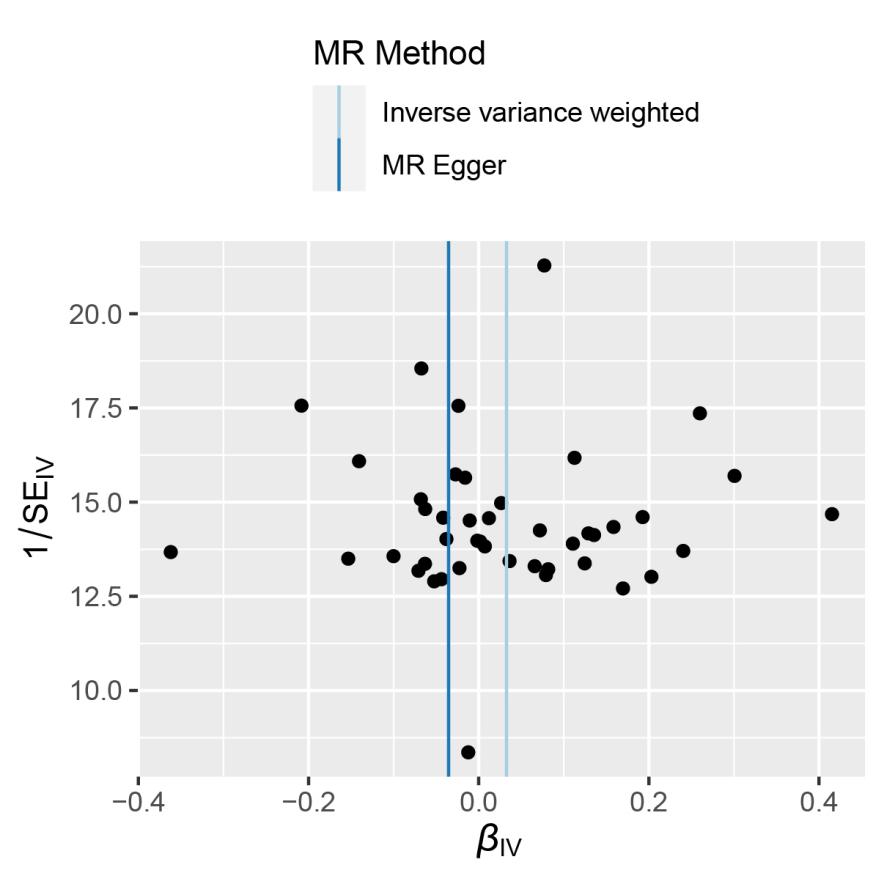


**Figure S12.** Funnel plot for MR analyses of causal effect of Bipolar disorder on Hemorrhoidal Disease


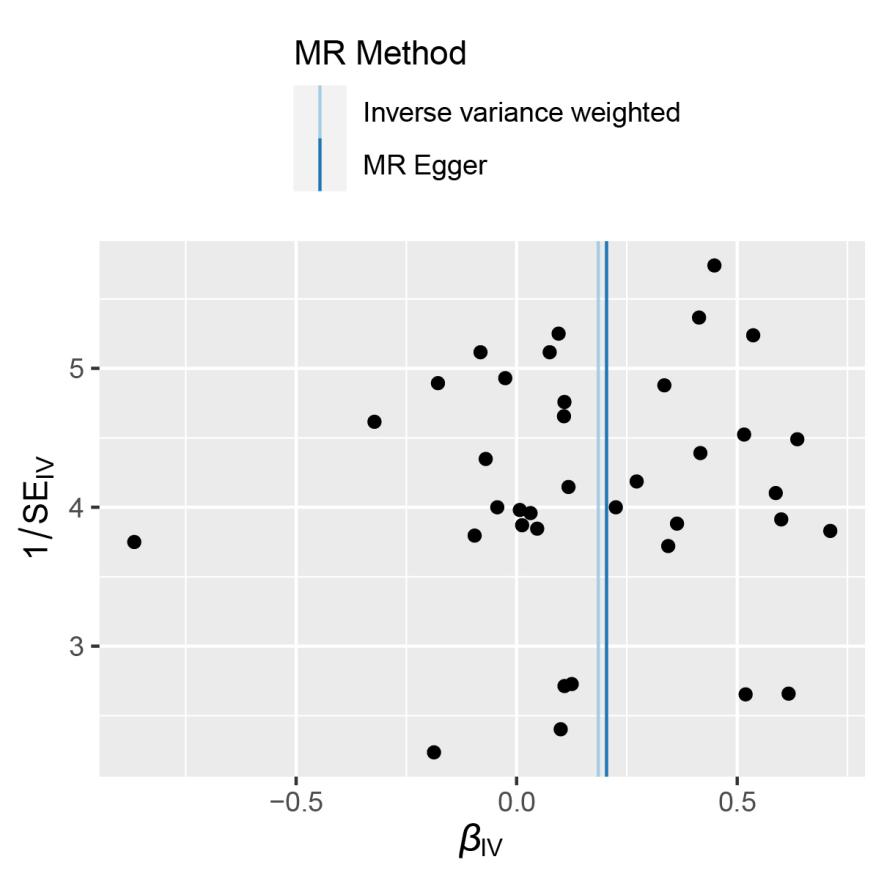


**Figure S13.** Funnel plot for MR analyses of causal effect of Depression on Hemorrhoidal Disease


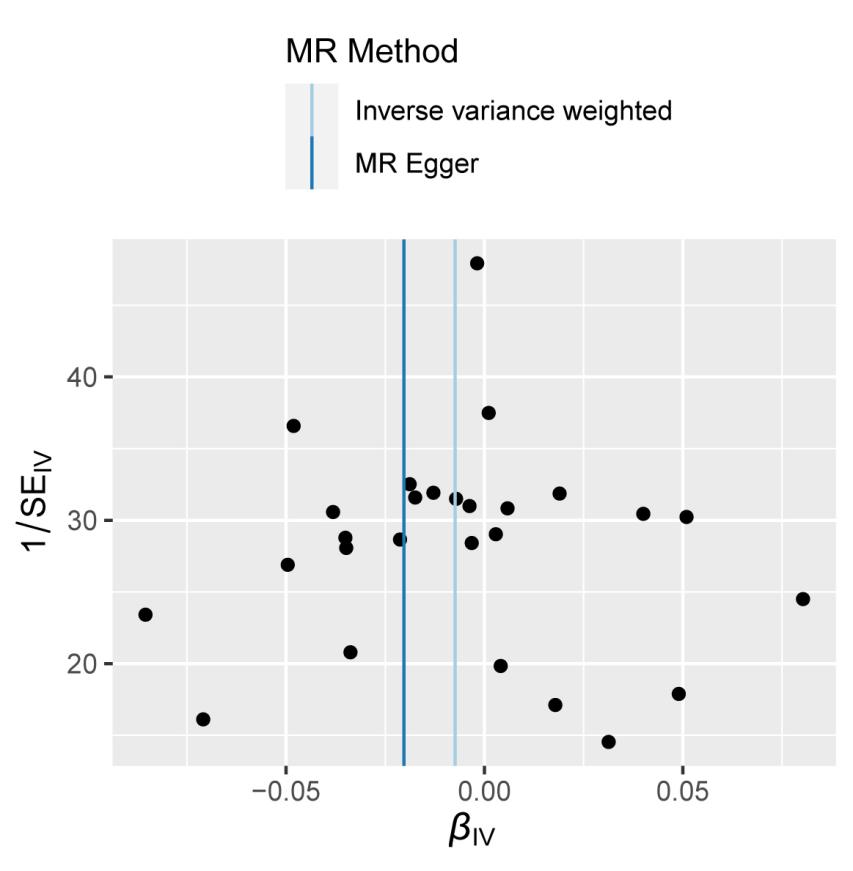


**Figure S14.** Funnel plot for MR analyses of causal effect of Schizophrenia on Hemorrhoidal Disease


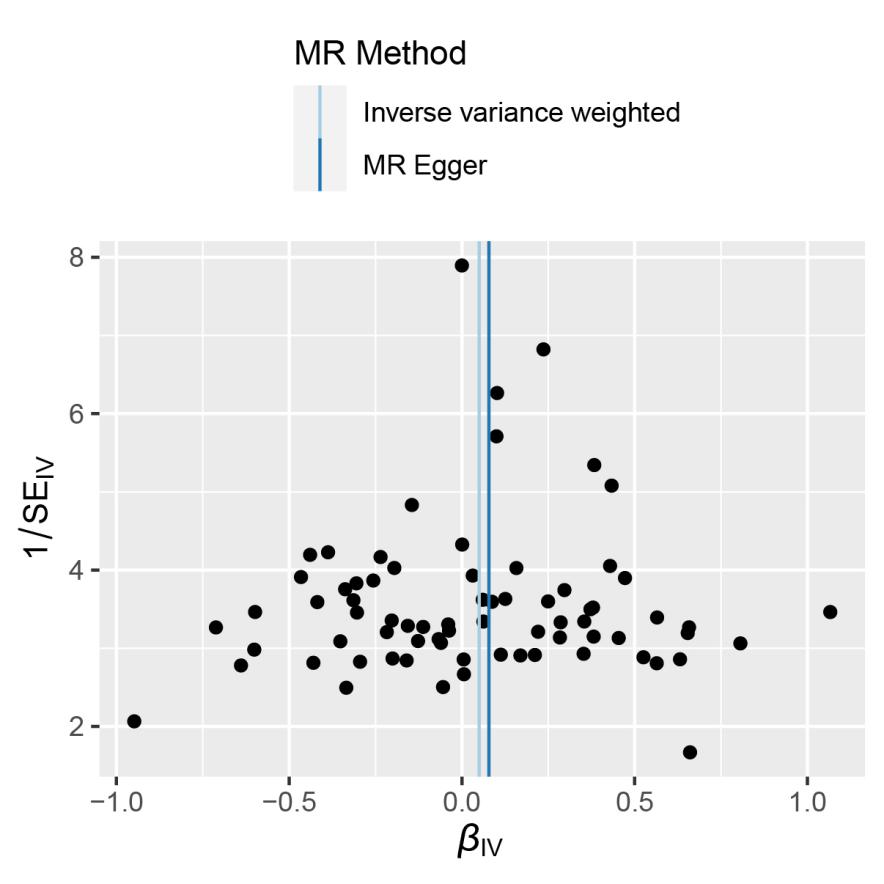


**Figure S15.** Funnel plot for MR analyses of causal effect of Hemorrhoidal Disease

on Anxiety disorders


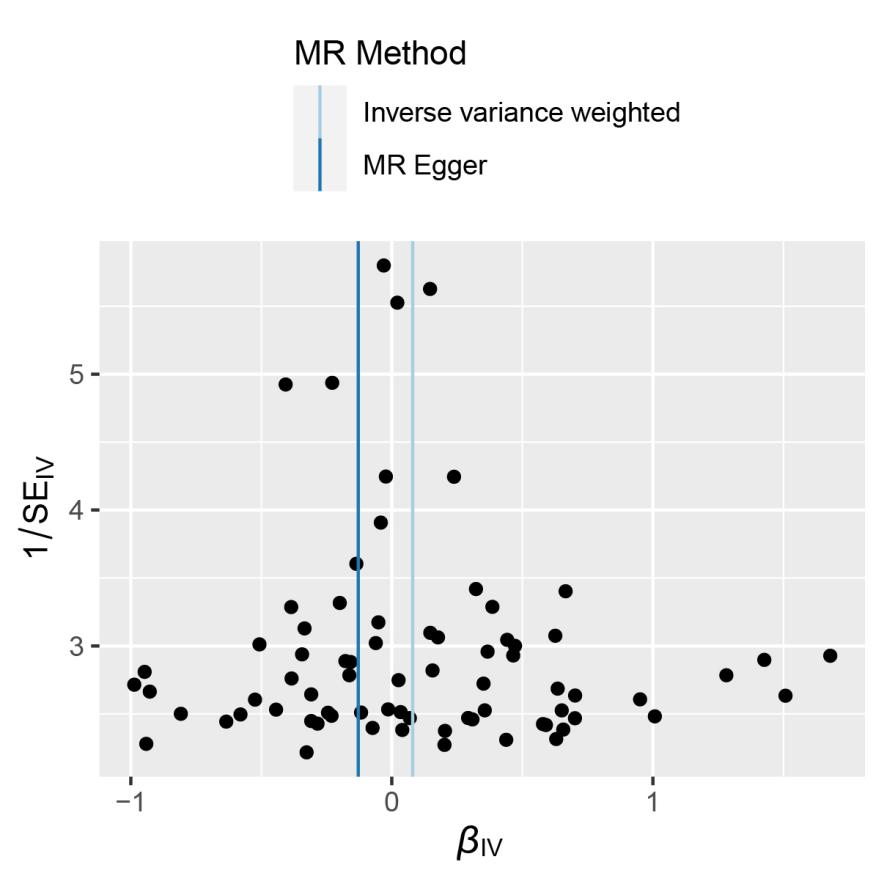


**Figure S16.** Funnel plot for MR analyses of causal effect of Hemorrhoidal Disease

on Bipolar disorder


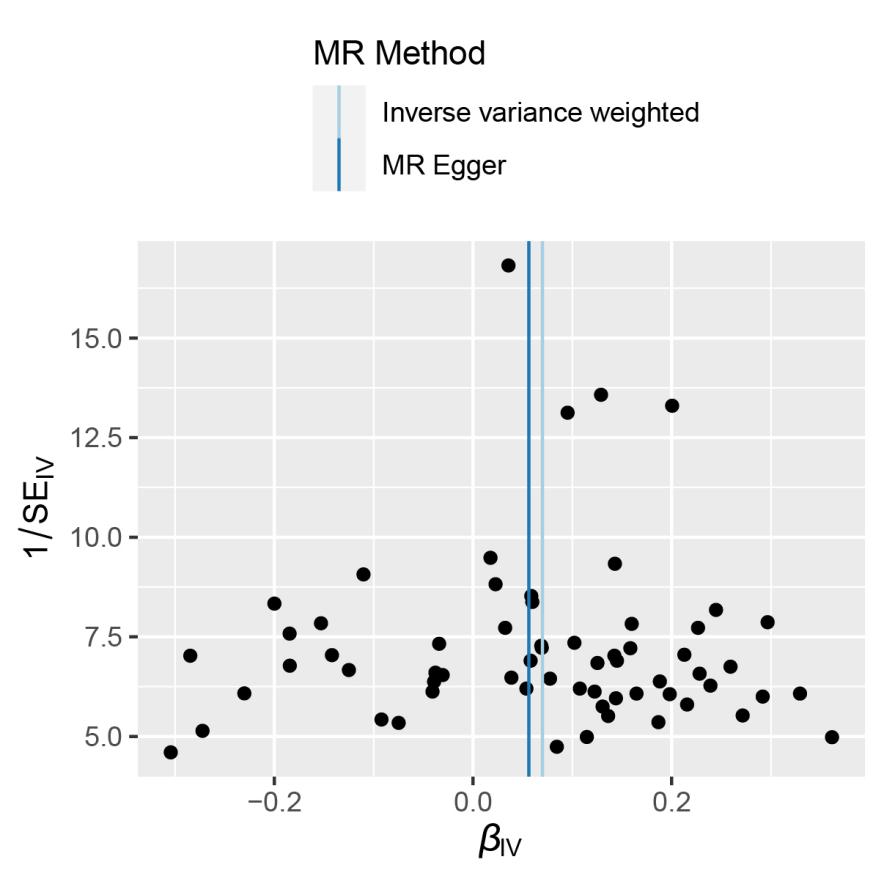


**Figure S17.** Funnel plot for MR analyses of causal effect of Hemorrhoidal Disease

on Depression


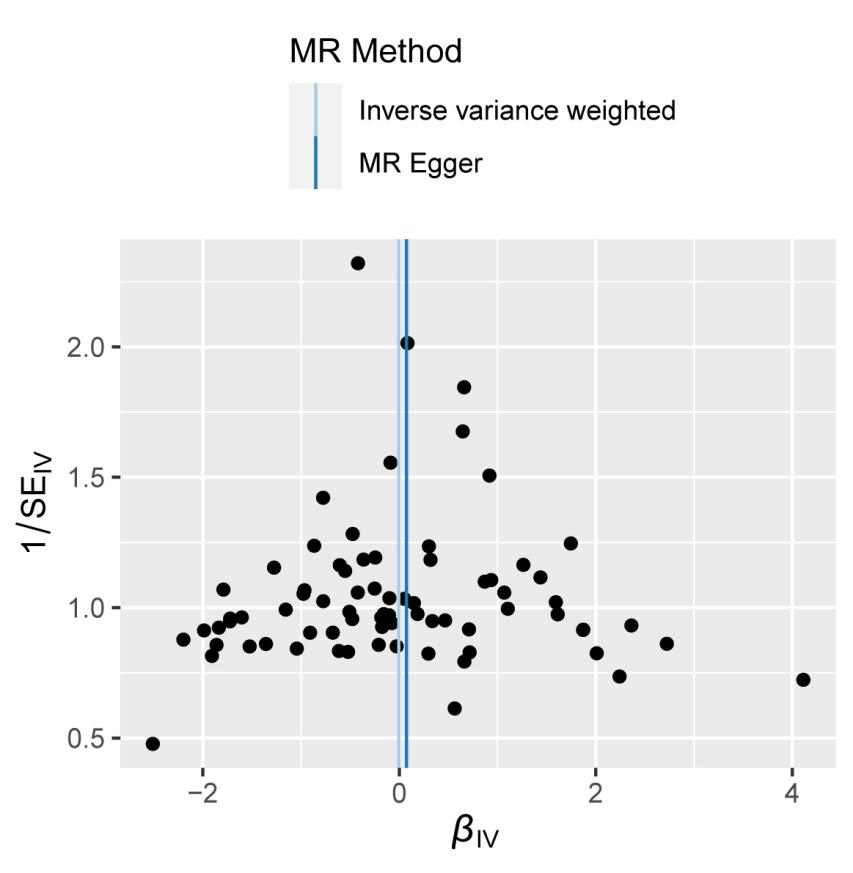


**Figure S18.** Funnel plot for MR analyses of causal effect of Hemorrhoidal Disease

on Schizophrenia
